# Sexually Antagonistic Selection in Human Male Homosexuality

**DOI:** 10.1371/journal.pone.0002282

**Published:** 2008-06-18

**Authors:** Andrea Camperio Ciani, Paolo Cermelli, Giovanni Zanzotto

**Affiliations:** 1 Dipartimento di Psicologia Generale, Università di Padova, Padova, Italy; 2 Dipartimento di Matematica, Università di Torino, Torino, Italy; 3 Dipartimento di Metodi e Modelli Matematici per le Scienze Applicate, Università di Padova, Padova, Italy; The University of New South Wales, Australia

## Abstract

Several lines of evidence indicate the existence of genetic factors influencing male homosexuality and bisexuality. In spite of its relatively low frequency, the stable permanence in all human populations of this apparently detrimental trait constitutes a puzzling ‘Darwinian paradox’. Furthermore, several studies have pointed out relevant asymmetries in the distribution of both male homosexuality and of female fecundity in the parental lines of homosexual vs. heterosexual males. A number of hypotheses have attempted to give an evolutionary explanation for the long-standing persistence of this trait, and for its asymmetric distribution in family lines; however a satisfactory understanding of the population genetics of male homosexuality is lacking at present. We perform a systematic mathematical analysis of the propagation and equilibrium of the putative genetic factors for male homosexuality in the population, based on the selection equation for one or two diallelic loci and Bayesian statistics for pedigree investigation. We show that only the two-locus genetic model with at least one locus on the X chromosome, and in which gene expression is sexually antagonistic (increasing female fitness but decreasing male fitness), accounts for all known empirical data. Our results help clarify the basic evolutionary dynamics of male homosexuality, establishing this as a clearly ascertained sexually antagonistic human trait.

## Introduction

### Background

The debate over the origin and evolutionary basis of human male homosexuality has attracted and continues to attract the attention of researchers and the public alike. One main source of interest is that various evidence collected in the last decades [1]–[14] strongly points to the existence of genetic factors influencing male homosexuality or bisexuality (GFMH). A number of genetic or familial studies have even attempted the identification of the loci related to the trait, with not yet conclusive results [1], [4], [5], [9], [14], [15]. While clarifying various aspects of the phenomenon, the evidence-based assumption of a genetic loading for homosexuality in males also generates a number of questions. One especially puzzling fact regards the long-standing persistence of this apparently detrimental trait, with the associated stability of polymorphic human populations; this is a possible ‘Darwinian paradox’: since male homosexuals don't mate with the opposite sex, shouldn't any ‘genes promoting homosexuality’ have died out of the population by now?

Several proposals have been advanced to explain the origin and permanence of male homosexuality from a genetic standpoint. Kin selection was earlier invoked [13], [16], [17], and later refuted [18], [19]. Other suggestions followed, the more recent debate being broadly focused on three lines of argument, not all based on genetic factors: *overdominance* (i.e. male heterozygous advantage, [7], [20]–[22]), *maternal effects* on male offspring (such as maternal selection [23], or maternal genomic imprinting [9]), and *sexually antagonistic selection* [2], [13], [15], [24]–[27]. Such proposals open many basic problems regarding the dynamics of the putative GFMH, and call for a satisfactory population genetic treatment of their propagation, which is lacking at present. This issue has been recently addressed [28], where a number of genetic models inspired by the above hypotheses (overdominance, maternal effects, sexual antagonism) were explored, assuming a single diallelic locus, either autosomal or X-linked, for the GFMH (see also [7], [29]).

The analysis in [28] characterizes the ranges of selection parameters, such as dominance or cost to males and gain to females, which guarantee the persistence of the trait in a population, with results that are in principle experimentally testable. However, a comparison with available data, which we perform here (see below), shows that one-locus models do not properly account for the observed GFMH dynamics. Most such models, indeed, are too unstable and cannot guarantee polymorphism under the normal variability of population conditions, such as average fecundities. These results may lead to speculate that the GFMH can easily invade a population, or, in contrast, die out of it, with the possible prediction of either widespread male homo- and bisexuality [30], or of a complete extinction of these characters.

### Empirical data: stability and pedigree asymmetries

The above conclusions indicate these models are inadequate for describing the known evidence on human male homosexuality. First, anecdotal accounts, whenever available, support the idea that homosexuality and bisexuality have always been present in past human populations; there are, furthermore, no records of populations with predominantly bisexual or homosexual male members. Second, all the present-day studies which have investigated male homosexuality in humans yield low frequencies of the order of a few percent of males in Western countries. While the historical record is clearly imprecise, and contemporary comparative studies may also indicate the (perceived) differences in social acceptance of homosexuality rather than true frequencies, it seems well established that no total extinction nor fixation of the genetic factors for male homosexuality have ever occurred, and that any GFMH would have always been present in (non-zero but) relatively low frequencies.

Other fundamental empirical observations connected to male homosexuality indicate the existence of characteristic ‘pedigree asymmetries’ concerning (a) male sexual orientation, and (b) female fecundity. Specifically, male homosexuality is higher in the maternal line of homosexuals, relative to all other parental lines of either homo- and heterosexuals ([2], [4], [10]–[13], [24], but see [1]). Recent research has also associated a higher female fecundity to male homosexuality: indeed, it was found that homosexuals' mothers are more fecund than mothers of heterosexuals [2], [6], [31]–[33]. Further female fecundity asymmetries include a higher fecundity of maternal vs. paternal aunts of homosexuals [2], [32], [33] (see also below).

To summarize, the effects observed in connection to human male homosexuality impose that any relevant population genetic models meet at least the following basic requirements: (A) *Stable gene polymorphism*, i.e., polymorphism should be maintained under a wide range of circumstances (such as variable average fecundities), and with relatively low frequencies; (B) *Pedigree asymmetries*, i.e., the models should also account for: (B1) the asymmetries in sexual preference, and (B2) the asymmetries in fecundity, observed in relation to this human trait.

In order to investigate the propagation and persistence of the GFMH, in this work we absolve a two-fold task. We first consider all the relevant population genetic models based on the hypothesis that these hereditary factors are localized on either one or two loci. We then discuss the compatibility of such models with the available empirical data, according to the requirements (A)–(B1)–(B2) above.

## Methods

### Population genetic models

By definition, the phenotypic expression of the GFMH affects the mating behaviour of both male and female carriers, so that we can assume that the spread of the factors in the population can be described by a fertility equation with multiplicative fitness (in what follows we use the terms ‘fitness’ and ‘fecundity’ with the same meaning). See the Document S1 for more details.

#### Direct selection

Assume that the GFMH are associated to either a single locus or multiple loci, with *N* female and *M* male genotypes, and let (ξ_1_,…, ξ*_N_*) be the proportions of the female genotypes 1,…, *N*, and (η_1_,…, η*_M_*) the proportions of the male genotypes 1,…, *M*, in the population at a given generation. For non overlapping generations and infinite population size, the fertility equation yields the genotype proportions (ξ′_1_,…, ξ′*_N_*) and (η′_1_,…, η′*_M_*) at the following generation:
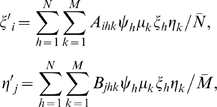
(1)with *N̅* and *M̅* normalizing factors, and where ψ*_h_* = *f_h_*/*f_N_*, μ*_k_* = *m_k_*/*m_M_* are the normalized fecundities of the female genotype *h* and the male genotype *k* respectively, with *f_h_* and *m_k_* the female and male fecundities. The product ψ*_h_*μ*_k_* may be interpreted as the mating probability of the genotypes *h* and *k*, while the coefficients *A_ihk_* and *B_jhk_* (listed in Document S1) are the conditional probabilities that a daughter/son of parents with genotype *h* and *k* has genotype *i* or *j*. Relation (1) is an iterative formula that allows to compute the evolution of the genotype proportions through generations given the initial distribution. If the genotype proportions approach values that remain fixed in the subsequent generations, we have an attracting equilibrium of (1); to find such equilibria, we iterate (1) numerically until convergence.

#### Maternal effects

The above formulation is based on the assumption that the mating behaviour of an individual is directly influenced by her/his genotype. An alternative model assumes that male fecundity is affected by maternal genotype only. The corresponding iterative formula is given in Document S1. The relevant parameters in this case are the female normalized fecundities ψ*_h_*, and the fitness μ*_h_*, of sons of mothers with genotype *h*.

#### Genomic imprinting

In this case, a particular allele is active in a son only if inherited from the mother, and male genotypes split according to the provenience of the gametes. For instance, in the case of a single autosomal locus, the male genotype *Aa* splits into the genotypes *A_m_a_p_* and *a_m_A_p_*. With this modification the iterative formula (1) may still be used.

## Results

### Specific models and results

The GFMH-carrying males (conventionally referred to as ‘homosexuals’) are assumed to exhibit behaviors that lower their average fecundity *m_GFMH_* as a population's subgroup, with respect to the average fecundity *m_b_* of non-GFMH-carrying males. In contrast, the GFMH is assumed to increase the average fecundity of female carriers to a value *f_GFMH_* greater than the baseline fecundity *f_b_* of non-carriers. Therefore, for all models discussed below, the parameters summarizing the main information on the fecundities of female and male carriers are respectively:
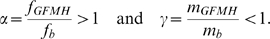
(2)


#### One-locus models

All one-locus models are diallelic with alleles *A* and *a*, with *A* the GFMH-associated allele. Assuming either dominance or overdominance in females, we study the following cases:

(1a) one autosomal locus with overdominance (increased heterozygote fitness) in both sexes:
(1b) one autosomal locus with overdominance in males and directional selection in females (male heterozygotes and female homo-heterozygotes have greater fitness);
(1c) one autosomal locus with sexually antagonistic selection (homozygosis increases female fitness, but decreases males fitness);
(2a) one X-linked locus with overdominance in females;
(2b) [(2c)] one X-linked locus with sexually antagonistic selection for an allele favoring females [males].
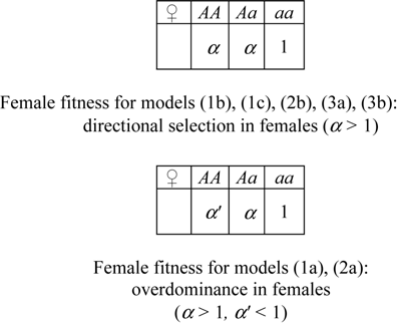

**Figure 1:**
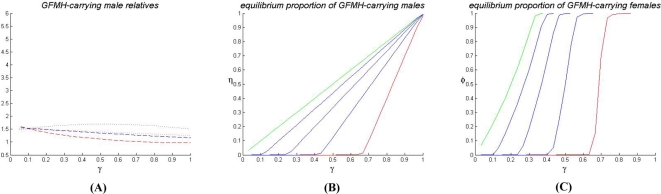
Some predictions of one-locus models. (A) Output (*ii*) for model (1b) - overdominance on autosome: sexual-orientation asymmetries, as functions of γ (for α = 1.4). Ratio between the predicted proportions of male homosexuals in the matriline vs. the patriline of homosexuals (red-dashed plot), and the same for heterosexuals (blue-dashed plot); ratio between the predicted proportions of male homosexuals in the matriline of homosexuals vs. the matriline of heterosexuals (red-dotted plot), and the same for the patriline (blue-dotted plot). (B) Output (*i*) for model (2b) - sexually antagonistic X-linked locus: equilibrium proportion η of homosexuals as a function of γ, for varying values of α (red plot: α = 1.2; green plot: α = 1.8; blue plots: 1.2<α<1.8). (C) Same output (*i*) for model (3a) - maternal selection on autosome.

Furthermore, we study the following models (3), which also include maternal effects [28]:

(3a) one autosomal locus with maternal selection on males and direct selection in females (male genotype is irrelevant for homosexuality, which is completely determined by the maternal genotype, and the genotype which induces homosexuality in sons is advantageous for the female;
(3b) one X-linked locus with maternal selection on males and direct selection in females.
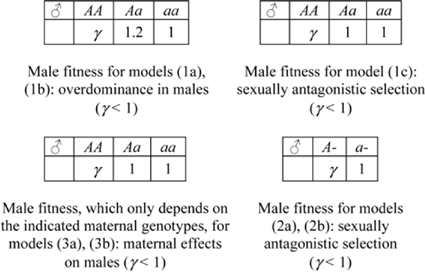

**Figure 2:**
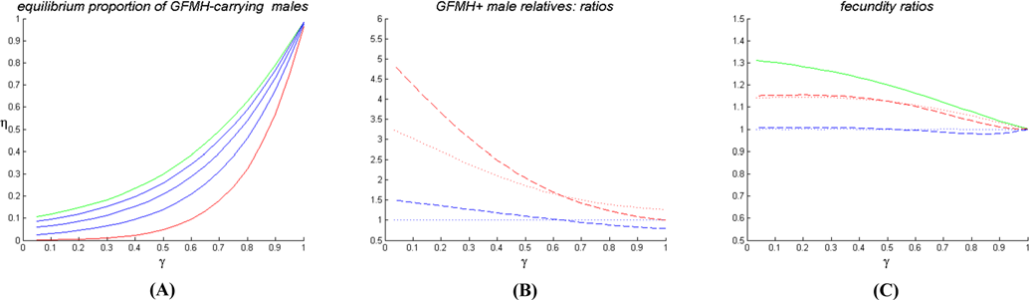
Predictions of model (4b) involving two X-linked loci with sexually antagonistic selection, for *u* = 1. (A) Output (*i*): equilibrium proportion η of GFMH-carrying males as a function of γ, for varying values of α (red plot: α = 1.2; green plot: α = 1.8; blue plots: 1.2<α<1.8). (B) Output (*ii*): pedigree asymmetries in male sexual orientation, as functions of γ (for α = 1.4). Ratio between the predicted proportions of male homosexuals in the matriline vs. the patriline of homosexuals (red-dashed plot), and the same for heterosexuals (blue-dashed plot); ratio between the predicted proportions of male homosexuals in the matriline of homosexuals vs. the matriline of heterosexuals (red-dotted plot), and the same for the patriline (blue-dotted plot). (C) Outputs (*iii*)–(*iv*): pedigree asymmetries in female fecundity, as functions of γ, for α = 1.4. Fecundity ratio of homosexuals' mothers to heterosexuals' mothers (green plot); fecundity ratio of maternal vs. paternal aunts of homosexuals (red-dashed plot), and the same for heterosexuals (blue-dashed plot); fecundity ratio between maternal aunts of homosexuals vs. maternal aunts of heterosexuals (red-dotted plot); the same for the paternal aunts (blue-dotted plot).

For each model we analyze, as a function of α and γ, the following outputs (see Document S1 for more details):

(*i*) The equilibrium frequency η of GFMH-carrying males
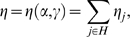
where *H* are the male genotypes associated to the GFMH. This parameter yields information on population-polymorphism stability under variations of the input parameters, see requirement (A). For the models based on maternal effects, we consider instead the equilibrium frequency φ of GFMH-carrying females
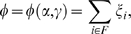
where *F* is the set of female genotypes associated to the GFMH.
(*ii*) The ratios between the proportions of male homosexuals in the parental lines of homosexuals and of heterosexuals (these outputs describe the sexual-orientation asymmetries related to requirement (B1));
(*iii*) The ratio between the fecundity of mothers of homosexuals and the fecundity of mothers of heterosexuals;
(*iv*) The fecundity ratios of maternal vs. paternal aunts of homosexuals, and of heterosexuals (outputs (*iii*)–(*iv*) describe the fecundity asymmetries related to requirement (B2)).

The correlation matrices for the pedigree analysis have been computed by using Bayes' theorem, which yields the conditional probabilities of parental genotypes given the offspring genotype. The relevant parameter ranges considered are as follows: the data in [2], [32], [33] give 0.2<γ<0.7 as a significant experimental window for the input γ. For the input α, the range 1.1<α<1.8 typically yields values of the outputs (*i*)–(*iv*) which are closest, in the best models, to the values reported in the experimental literature.

#### Results of one-locus models

The complete study of the above one-locus models, which can be found in the Document S1, shows they are all incompatible with either requirement (A) or (B) or both. Fig. 1 summarizes some results: we plot, for three representative one-locus models, the outputs which do not meet either condition (A) or (B). Specifically, in Fig. 1A we show the output (*ii*) for model (1b), referring to sexual-orientation asymmetries. Contrary to requirement (B1), these graphs do not account for the higher frequency of homosexuality in the matriline of homosexuals relative to all other parental lines [2], [4], [10]–[12], [24], [32], [33]. In Figs. 1B–1C we show the output (*i*) for models (2b) and (3a) respectively. In both cases requirement (A) on polymorphism stability is not met, as small variations of the input parameters leads to fixation or extinction of the GFMH. Model (1c) is not adequate for the same reasons as model (1b). Model (2c) is unstable as model (2b), because it is formally equivalent to it upon allele interchange. Model (3b) is unstable as model (3a). Finally, the remaining models (1a) and (2a) had already been deemed not suitable in [28].

**Figure 3:**
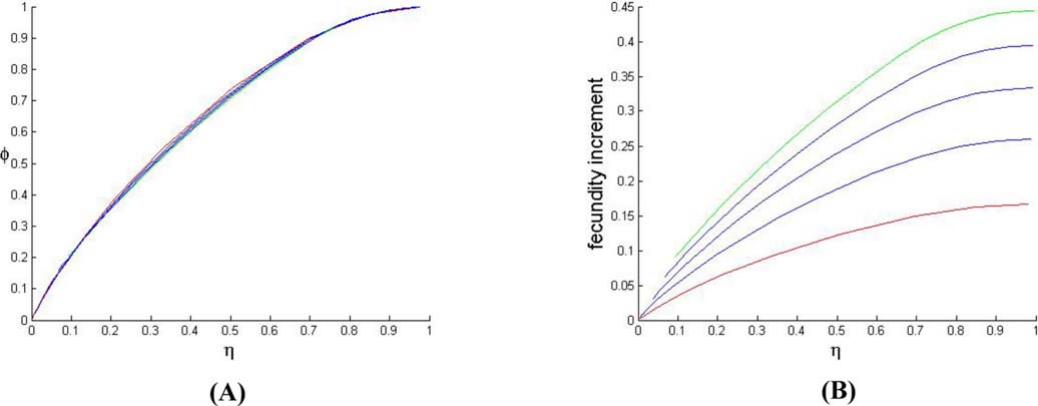
Equilibrium frequencies and total fecundity increase due to the GFMH. (A) Predicted correlation, under varying γ, between the equilibrium proportion φ of GFMH-carrying females and the equilibrium proportion η of GFMH-carrying males in the population, for model (4b) - sexually antagonistic two X-linked loci. Plots are at constant α. (B) Predicted total fecundity increment Δ*f* in the population at equilibrium due to the presence of the GFMH, as a function of the equilibrium proportion η of homosexuals. Plots are at constant α (red plot: α = 1.2; green plot: α = 1.8; blue plots: 1.2<α<1.8).

#### Two-locus models

We investigate the following diallelic two-locus models (alleles denoted by *A,a* and *B,b*):

(4) one X-linked locus (alleles *A,a*) together with: either (4a) one autosomal locus, or (4b) another X-linked locus, with sexually antagonistic selection;
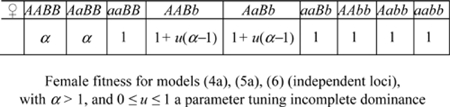


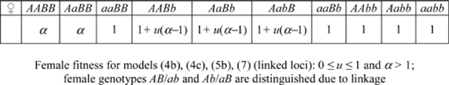


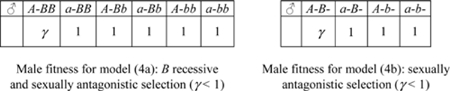



(4c) two autosomal loci with sexually antagonistic selection;
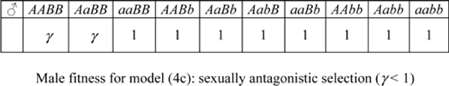



(5) one autosomal locus (alleles *B,b*) together with: either (5a) one X-linked locus, or (5b) another autosomal locus, with overdominance in males and directional selection in females (male heterozygotes and female homozygotes have greater fitness);
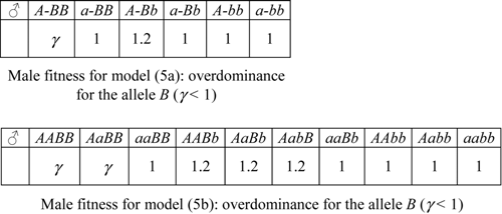



(6) two independent autosomal loci with maternal genomic imprinting of one locus. Assuming for instance that the gamete *A* is subject to imprinting and is active only when inherited by the mother, we split the male genotype AB/aB in two classes: AB(maternal)/aB(paternal) and aB(maternal)/AB(paternal)). Also, we assume that GFMH-related allele *B* is recessive in females, and selection is sexually antagonistic;
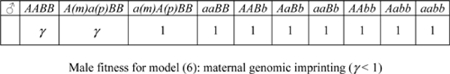



(7) two autosomal loci with maternal selection on males and direct selection on females.
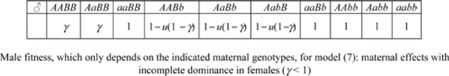



We remark that the above list includes all the two-locus models for which the GFMH have the following properties:

- the GFMH are expressed by the presence of at least an ‘activator allele’ *A* on one locus, which is necessary for the expression of another ‘trait-promoting allele’ *B*. The allele *A* is therefore always dominant. Furthermore, the dominant allele *A* always occupies the X-linked locus when at least one such locus is available in any given model;
- the general case of incomplete dominance is considered for the allele *B*, in the sense that a parameter *u* modulates the effect on the fitness in each sex when heterozygous for *B* (so that *B* is ‘recessive’ for *u* = 0, and *B* is ‘dominant’ for *u* = 1). However, we have only considered models in which the GFMH are expressed in males when at least a copy of the activator allele *A* is present on one locus, together with two copies of the allele *B* on the other locus (one copy for model 4b); i.e. *B* is always recessive (*u* = 0) in males when *B* is autosomal.

Then, sexually antagonistic selection is considered through models (4), while overdominance in males, described by assigning an increased fitness to males heterozygous for the allele *B* residing on the autosome, is considered through models (5). In model (6), which we consider only for independent loci on distinct autosomes, the activator allele *A* is expressed in males only when inherited from the mother, so as to mimick maternal genomic imprinting effects such as those envisaged in [9] for a multi-locus GFMH. Finally, genetic maternal effects are described through model (7).

We have considered explicitly only the two-locus models satisfying to assumptions (*a*) and (*b*) above because the numerical tests on models in which such conditions do not hold (i.e., in which the allele *A* is recessive, or in which *B* is not recessive in males, or in which the dominant allele *A* is autosomal and *B* is X-linked), all give results which are worse, in meeting requirements (A)–(B1)–(B2), than the models listed above, especially as they fail to satisfy request (A) on polymorphism stability. See more on dominance below.

#### Results of two-locus models

For the two-locus models in the previous list, we have assessed the compatibility of the outputs (*i*)–(*iv*) with requirements (A)–(B1)–(B2) for varying α and γ. The results show that the models involving only autosomal loci are not adequate, as (7) is unstable, and (4c), (6) cannot explain sexual-preference asymmetries (see Document S1 for details). The latter are also not accounted for by any of the overdominance models (5). Models (4), however, in which the GFMH are sexually antagonistic and related to one or more X-linked loci, are consistent with both the constraints (A) and (B1)–(B2). The best qualitative fit with the experimental data is given by model (4b), whose outputs are shown in Fig. 2. We observe (Fig. 2A) the stability of the trait at low frequencies, with no GMFH extinction or fixation ever predicted, even for large variations in the input fecundities α and γ. The required sexual-orientation asymmetries are reproduced, too, as a higher frequency of homosexual males in the maternal line of homosexuals is obtained compared to all other parental lines (Fig. 2B), the quantitative fit to the data depending on the values of α and γ. Also the fecundity asymmetries are well accounted for, as the mothers of homosexuals have higher fecundity than the mothers of heterosexuals, in a range which includes the empirically-observed ratio 1.2 (see [2]). Moreover, the fecundity is higher in maternal aunts than in paternal aunts of homosexuals (Fig. 2C). The phenomenon is reversed in heterosexuals, for which fecundity is higher in paternal rather than in maternal aunts. Remarkably, both models (4a) and (4b) show this effect, giving a higher fecundity of paternal aunts in heterosexuals (see Fig. 2C and Document S1). This prediction was confirmed by re-analyzing the empirical data in [2], [32] [33], with an even more marked effect than predicted, and which the model can better fit by tuning the incomplete dominance parameter *u*. Models (4) also give a higher expected fecundity in homosexuals' grandmothers, relative to heterosexuals', but significant data for validation are lacking.

## Discussion

Our analysis allows us to draw several conclusions that clarify the basic evolutionary dynamics of the genetic factors influencing human male homosexuality and the related female fecundity increase, resolving a number of open questions. As a main point, we can exclude the GFMH propagation mechanisms based on overdominance (male heterozygote advantage), because none of the models (1b), (5a), (5b) account satisfactorily for the sexual-orientation asymmetries of requirement (B1). At this level of genetic analysis, we can also exclude maternal effects, including maternal genomic imprinting, as they lead too easily to GFMH extinction or fixation, against requirement (A). Only the hypothesis that the GFMH are characterized by sexually antagonistic selection (i.e. the GFMH favor one sex and disfavor the other) produces viable population genetic models (see the case (4) above) leading to the persistence of the trait at low frequencies and capable of accounting for the related pedigree asymmetries. For this reason, predictions of possible widespread diffusion of male homo- or bisexuality in human populations [30] are not warranted, as stable low levels of this character are actually compatible with a broad range of parameters in population genetic models.

The fact that both the models (4a) and (4b), and only those, fit qualitatively the available empirical data not only establishes the sexually antagonistic character of this human trait, but also indicates the presence of at least one X-linked locus for the GFMH. This agrees with the relation between X-linkage of the GFMH and sexual antagonism also pointed out in [28]. The best qualitative agreement with the data is obtained through model (4b) with two X-linked loci: the subtleties of the observed asymmetries therefore indicate the genetics and inheritance dynamics of the GMFH to be modulated by an X-linked switch activating a further locus on the sexual chromosome, possibly together with other autosomal components [9] not identifiable through our analysis.

The two best models (4a) and (4b) allow us to draw a number of conclusions regarding the dominance for the alleles involved in the GFMH. We recall that in both models the allele *A*, which resides on the X chromosome, is dominant (this is indeed the case in all the other models considered above, for otherwise the stability of polymorphism is not guaranteed). The numerical simulations show, as general trends, that in both models the dominance of *B* in females (i.e. high values of *u*) improves polymorphism stability, while the localization of the GFMH entirely on X-linked loci gives qualitatively better pedigree asymmetries, as can be intuitively expected, if compared to a GFMH partially residing on the autosome. In detail, for model (4a) involving an autosomal *B*, the best stability is obtained when for *u* = 1. However, this case does not produce the correct pedigree asymmetries; small or intermediate values of *u*, giving almost unaffected fitness to females heterozygous for *B*, are optimal to satisfy qualitatively all conditions (A)–(B1)–(B2); see Fig. 5 in Document S1. Therefore in model (4a) the allele *B* should be almost ‘recessive’ in females (recall it is always recessive in males). Also when *B* is on the X chromosome (model 4b), the best conditions for polymorphism stability are given by *u* = 1, i.e. when females who are homo- or heterozygous for *B* have the same fitness. However, in this case both the GFMH loci are X-linked, and the pedigree asymmetries result to be only slightly affected by the parameter *u*, so that the requirements (A)–(B1)–(B2) are qualitatively best satisfied for *u* = 1 (see Fig. 2 above and Fig. 6 in Document S1). We conclude that in model (4b) the allele *B* should be dominant in females (while it is still recessive in males).

We notice that model (4b) also predicts a higher concordance in sexual orientation between biological brothers than model (4a); further information on the absolute values of frequency of homosexuality, or direct gene investigation, which are unavailable at present, will help to discriminate between the two possibilities. Closer quantitative adherence to the experimental data can in principle be obtained by increasing the number of loci related to the GFMH. Such more complex modeling however should not change the basic insight provided by the simplest two-locus approach investigated here.

Our results, which exclude both overdominance and maternal effects on male offspring, also point to a likely scenario of *androphilic* phenotypic expression of the GFMH, i.e., an expression that specifically increases the attraction to males in both sexes, rather than inducing a more general phenotipic feminization. Androphilia is indeed consistent in a more natural way with the sexually antagonistic hypothesis, in contrast to the hypothesis of feminizing GFMH or maternal GFMH, which are better associated to the genetic models based respectively on overdominance in males, or on genetic maternal effects, which we considered above. See the remarks on phenotypic expression in Document S1 for more details. We notice that the conclusion of an androphilic effect of the GFMH in principle allows one to make testable predictions regarding the behavior of GFMH carriers, along the lines for instance of [36], [37].

Sexually antagonistic selection has been considered in the past [38]–[40], although its role in evolutionary processes has been generally underestimated; it has however recently received both theoretical and empirical attention due to its potential ubiquity in dioecious species [27], [41]–[43]. Sexually antagonistic selection is at present recognized as a powerful mechanism through which genetic variation of fitness is maintained despite sexual selection in biological populations, in insects [42], [44], [45], birds [42], [46], and mammals [43], leading to population divergence and possibly speciation [41], [44], [47]–[50]. Our findings firmly establish, with a particularly relevant example, the occurrence of sexually antagonistic characters in humans. This point of view may help shift the focus away from male homosexuality *per se*: rather than concentrating on the sole aspect of the reduced male fecundity that it entails, we can place it within the more general framework of a genetic trait with gender-specific benefit, which may have evolved by increasing the fecundity of females. A consequence of this is that the entire population exhibits a high fecundity variation, and, as we show, the trait can neither disappear nor completely invade the gene pool. Indeed, the GFMH may belong to a possibly wide, but at present still poorly understood, class of sexually antagonistic characters that contribute to the maintenance of the observed genetic variation in human populations. As such characters are mostly expected to have a sex-linked component, the present treatment of the GMFH should provide basic understanding also of the dynamics of any such general sexual antagonistic traits.

While the latter are generally assumed to favor males and penalize females (but see [51]), we point out a counterintuitive implication of the presence of traits which increase female fecundity, as the sexually antagonistic GFMH. Fig. 3A shows that, at equilibrium, the proportion φ of GFMH-carrying females in the population positively correlates with the proportion η of GFMH-carrying males. Both φ and η affect the population's overall fecundity at equilibrium (see Document S1). Consider now the variation Δ*f* of the total fecundity due to the presence of the GFMH in a population at equilibrium (with respect to the population's baseline fecundity in the absence of GFMH):
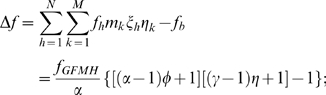
(3)we have that when *f_GFMH_* is constant, Δ*f* is a function of α and γ only. As the normalized fecundity α of GFMH-carrying females is inversely proportional to the baseline fecundity *f_b_* of non-GFMH-carrying females, a decrease of *f_b_* (due, for instance, to social or economic factors) results in a decrease of the total expected fitness in the population, but also in an increase of α. From (3), we find that Δ*f* is a positive and monotonically increasing function of both the variables α and η. This is shown in Fig. 3B, where we also see that the higher α, for given η, the larger is Δ*f*. We thus have the following consequences: (*a*) in a given population (α and η fixed) the presence of the GFMH always induces a *positive* increment Δ*f* of the total fecundity, with respect to the baseline value in the absence of the GFMH; (*b*) all else being the same, a higher proportion of homosexuals in a population indicates a comparatively higher total fecundity increment Δ*f*; (*c*) if due to external conditions the population's baseline fecundity is falling (which results in an increase of the fecundity α of GFMH-carrying females relative to the baseline), the increment of the population's fecundity Δ*f* due to the presence of the GFMH becomes proportionally more pronounced, mimicking a ‘buffer effect’ on any factors inducing the total fecundity decrease.

## Supporting Information

Document S1 (0.71 MB PDF)
