# Supplementary material for: Sexually Antagonistic Selection in Human Male Homosexuality
Source: PLoS One. 2008 Jun 18;3(6):e2282. doi: 10.1371/journal.pone.0002282 (PMC2427196; doi:10.1371/journal.pone.0002282)
Supplement: Document S1 — (0.71 MB PDF) [file pone.0002282.s001.pdf]

Supporting Information

for

'Sexually antagonistic selection in human male homosexuality'

by

A. Camperio Ciani, P. Cermelli and G. Zanzotto

1. *The selection equation*

1.1 Random mating with direct selection

For either a single or multiple locus system with N female genotypes and M male genotypes, let x_i and y_j (i=1,...,N, j=1,...,M) be respectively the female and male genotype proportions in the population. We assume that the phenotypic expression of the GFMH affects the fitness of carriers, viewed either as mating success (i.e., the probability of entering the mating pool) or as fecundity (the contribution to the total number of offspring) – which are equivalent under the hypothesis of random mating (see below formula (4)). Denote by f_i the fitness of a female with genotype i, by m_j the fitness of a male with genotype j, and by

$$\psi_i = f_i/f_N, \qquad \mu_j = m_j/m_M \,, \tag{1}$$

the corresponding normalized fitness (referred to as normalized *fecundity* in the main text). Assume first that fitness is interpreted as mating success, i.e., that it is proportional to the probability of entering the mating pool, and mating is random among the individuals in the pool. Then, for non-overlapping discrete generations and infinite population size, the genotype frequencies x'_i and y'_j at the next generation are given by the iterative relations (see [34]):

$$x'_i = \psi_i \sum_{h=1}^{N}\sum_{k=1}^{M} A_{ihk} x_h y_k \,/\, E(\psi), \qquad y'_j = \mu_j \sum_{h=1}^{N}\sum_{k=1}^{M} B_{jhk} x_h y_k \,/\, E(\mu), \tag{2}$$

where A_{ihk} and B_{jhk} are the conditional probabilities that a daughter/son of parents with genotypes h and k has genotype i or j, i.e.:

$$A_{ihk} = \Pr(d=i \mid m=h, f=k), \qquad B_{jhk} = \Pr(s=j \mid m=h, f=k)$$

(m = mother, f = father, d = daughter, s = son), and

$$E(\psi) = \sum_{i=1}^{N}\sum_{h=1}^{N}\sum_{k=1}^{M} \psi_i A_{ihk} x_h y_k \,, \qquad E(\mu) = \sum_{j=1}^{M}\sum_{h=1}^{N}\sum_{k=1}^{M} \mu_j B_{jhk} x_h y_k \,.$$

By setting

$$\xi_i = \frac{x_i/\psi_i}{\sum_{h=1}^{N} x_h/\psi_h}, \qquad \eta_j = \frac{y_j/\mu_j}{\sum_{k=1}^{M} y_k/\mu_k} \,, \tag{3}$$

for the proportions of female and male genotypes in the population before selection occurs, i.e. before entering the mating pool, we may rewrite the selection equation (2) in terms of ξ_i and η_j :

$$\xi_i' = \sum_{h=1}^{N}\sum_{k=1}^{M} A_{ihk}\psi_h\mu_k\xi_h\eta_k \,/\, \overline{N}, \qquad \eta_j' = \sum_{h=1}^{N}\sum_{k=1}^{M} B_{jhk}\psi_h\mu_k\xi_h\eta_k \,/\, \overline{M}, \tag{4}$$

with $\overline{N}$ and $\overline{M}$ suitable normalizing factors. The iterative formula (4) has the form of a fertility equation [35], and corresponds to random mating with multiplicative fertilities $\psi_h\mu_k$ for the mating between genotypes h and k. Hence, in view of (4), the fitness of each genotype may be also interpreted as its fecundity, i.e. the contribution of that genotype to the total number of offspring .

The coefficients A_{ihk} and B_{jhk} can be characterized in terms of the gamete-genotype correlation matrices as follows. Since for mixed or X-linked loci fathers may produce two types of gametes, according to the presence or the absence of the X chromosome, we label gametes with the X chromosome (female gametes) by the index $\alpha = 1,...,n$, and gametes containing the Y chromosome (male gametes) by the index $\beta = 1,...,m$. Define

$C_{\alpha h}$ = Pr(female gamete = α | maternal genotype = h),
$D_{\alpha k}$ = Pr(female gamete = α | paternal genotype = k),
$E_{\beta k}$ = Pr(male gamete = β | paternal genotype = k),

the matrices that give the correlation between gametes and parental genotypes, and let

$F_{i\alpha\alpha'}$ = Pr(daughter genotype = i | maternal gamete = α, paternal gamete = α'),
$G_{j\alpha\beta}$ = Pr(son genotype = j | maternal gamete = α, paternal gamete = β),

be the matrices correlating offspring genotypes and parental gametes. Then

$$A_{ihk} = \sum_{\alpha=1}^{n}\sum_{\alpha'=1}^{n} F_{i\alpha\alpha'}C_{\alpha h}D_{\alpha' k}, \qquad B_{jhk} = \sum_{\alpha=1}^{n}\sum_{\beta=1}^{m} G_{j\alpha\beta}C_{\alpha h}E_{\beta k}.$$

As is well known, the iterative system (2) yields a corresponding evolution equation for the gamete proportions

$$p_\alpha = \sum_{h=1}^{N} C_{\alpha h}x_h, \qquad q_{\alpha'} = \sum_{k=1}^{M} D_{\alpha' k}y_k, \qquad r_\beta = \sum_{k=1}^{M} E_{\beta k}y_k,$$

which we do not use here. The explicit form of the gamete-genotype correlation matrices for each model is given in Section 7.

1.2 Maternal effects

A simple way of accounting for maternal effects is by assuming that the maternal genotype affects sons' fitness regardless of their genotype [28]. Denoting by ρ_h, with $h=1,...,N$, the male fitness reduction due to maternal effects, the evolution equations for the genotype proportions are now

$$x_i' = \psi_i \sum_{h=1}^{N}\sum_{k=1}^{M} A_{ihk}x_h y_k \,/\, \hat{N}, \qquad y_j' = \sum_{h=1}^{N}\sum_{k=1}^{M} B_{jhk}\rho_h x_h y_k \,/\, \hat{M}, \tag{5}$$

with obvious definitions of the normalizing factors $\hat{N}$ and $\hat{M}$.

1.3 Genomic imprinting

In this case, the assumption is that a particular allele is active in a male only when inherited by the mother. To account for this mechanism, we enlarge the set of male genotypes, distinguishing two male genotypes according to the provenience of the gametes. For instance, in the case of a single autosomal locus, the male genotype *Aa* would split into the genotypes $A_m a_p$ and $a_m A_p$. With this modification, the selection equation in the form (4) may still be used.

2. *Description of the models*

A model based on the selection equation (2) is completely characterized by the correlation matrices $C_{\alpha h}$, $D_{\alpha k}$, $E_{\beta k}$, $F_{i\alpha\alpha'}$, $G_{j\alpha\beta}$, listed in Section 7 (which for multi-locus models depend on the recombination fraction *r*), and the values of the normalized fecundities ψ_i, μ_j, and ρ_i.

Specifically, we assume that the effect of the GFMH is to lower the average fecundity of male carriers, normalized with respect to non-GFMH-carrying males, to a value $\gamma < 1$ (except for the overdominance cases in which heterozygous GFMH-carrying males have average fecundity $\gamma' > 1$). In contrast, the GFMH is assumed to increase the average fecundity of female carriers to a value f_{GFMH} greater than the baseline fecundity f_b of female non-carriers. This is equivalent to setting the normalized fecundity α of GFMH-carrying females at a value $\alpha > 1$ (see formula (6) below). To allow for incomplete dominance, we assign to the fecundity of selected female heterozygotes an intermediate value between f_{GFMH} and f_b. The results of all models below will be discussed in terms of their dependence on the two main input parameters

$$\alpha = \frac{f_{GFMH}}{f_b} \quad \text{and} \quad \gamma\,, \tag{6}$$

We remark that the assumption that male GFMH-carriers have a given average fecundity $\gamma < 1$ is compatible with an array of individual behaviours that may be related to homosexuality, bisexuality, or even heterosexuality: the individual fecundity of GFMH-carrying males may thus take any positive value, under the constraint that the normalized average fecundity of male carriers be $\gamma < 1$. (When the distribution of individual normalized fecundities in the set of GFMH-carrying males clusters around the values 0 and 1, the value $1-\gamma$ can be interpreted as penetrance.)

The labelling of the models below is the same used in the main text. All one-locus models are diallelic with alleles *A* and *a*, with *A* the ‘trait-promoting allele’. Two-locus models are also diallelic (*A,a,B,b*), and we have focused on situations in which the allele *A* acts as an ‘activator allele’ whose presence is necessary to the expression of another ‘trait-promoting allele’ allele *B*, under the dominance assumptions discussed in the main text.

Remark on notation. When there is no ambiguity, for brevity hereafter we use, in lists of two-locus genotypes, an abbreviated notation in which the first two symbols in a string refer to the first locus and the second two symbols refer to the second locus, i.e. *Ab/ab* is denoted *Aabb*. We will also write GFMH+ and GFMH– to indicate GFMH-carriers and non-GFMH-carriers, respectively.

2.1 A single autosomal locus – models (1a), (1b), (1c), (3a)

In models (1a), (1b) and (1c), we have N=M=3, n=m=2, and i,j,h,k=1,2,3, label male and female genotypes (1=AA, 2=Aa, 3=aa), while α,β = 1,2, label male and female gametes (1=A, 2=a). Specifically, we have considered the following cases:

– Model (1b): one autosomal locus with overdominance in males and complete dominance in females (which results in directional selection in females)

$$\psi_1 = \psi_2 = \alpha > 1\ , \quad \psi_3 = 1, \qquad \mu_1 = \gamma < 1, \quad \mu_2 = \gamma' = 1.2, \quad \mu_3 = 1.$$

– Model (1c): one autosomal locus with sexually antagonistic selection for a recessive allele A in males

$$\psi_1 = \psi_2 = \alpha > 1, \quad \psi_3 = 1, \qquad \mu_1 = \gamma < 1, \quad \mu_2 = 1, \quad \mu_3 = 1.$$

– Model (3a): one autosomal locus with maternal effects on males and directional selection in females, see equation (5), again assuming complete dominance in females

$$\psi_1 = \psi_2 = \alpha > 1\ , \quad \psi_3 = 1, \qquad \rho_1 = \gamma < 1, \quad \rho_2 = 1, \quad \rho_3 = 1.$$

2.2 A single X-linked locus – models (2a), (2b), (3b)

Here N=3, M=2, n=2, m=1. Now i,j=1,2,3, label female genotypes (1=AA, 2=Aa, 3=aa), while h,k=1,2 for male genotypes (1=A-, 2=a-). Also, α=1,2, label female gametes (1=A, 2=a), and β=1 labels the male gamete (1=-). Among models (2a), (2b), (3b), we have studied only the two relevant cases:

– Model (2b): one X-linked locus with sexually antagonistic selection for a dominant allele in females

$$\psi_1 = \psi_2 = \alpha > 1\ , \quad \psi_3 = 1, \qquad \mu_1 = \gamma < 1, \quad \mu_2 = 1.$$

– Model (3b): one X-linked locus with maternal effects on males and directional selection in females, see equation (5), with complete dominance in females

$$\psi_1 = \psi_2 = \alpha > 1, \quad \psi_3 = 1, \qquad \rho_1 = \gamma < 1, \quad \rho_2 = 1, \quad \rho_3 = 1.$$

2.3 One autosomal locus (alleles *B,b*) and one X-linked locus (alleles *A,a*) – models (4a), (5a)

Here N=9, M=6, n=4, m=2. Now i,j=1,…,9, label female genotypes (1=$AABB$, 2=$AaBB$, 3=$aaBB$, 4=$AABb$, 5=$AaBb$, 6=$aaBb$, 7=$AAbb$, 8=$Aabb$, 9=$aabb$), while h,k=1,…,6, for male genotypes (1=A-BB, 2=a-BB, 3=A-Bb, 4=a-Bb, 5=A-bb, 6=a-bb). Also, α=1,…,4, label female gametes (1=AB, 2=aB, 3=Ab, 4=ab), and β=1,2 label the male gametes (1=B-, 2=b-).

Assuming the general case of incomplete dominance for the allele B in females, we write

$$\psi_1 = \psi_2 = \alpha > 1\ , \qquad \psi_4 = \psi_5 = 1 + u(\alpha - 1), \qquad \psi_3 = \psi_6 = \psi_7 = \psi_8 = \psi_9 = 1,$$

where hereafter the parameter u, with $0 \le u \le 1$, is a parameter tuning the incomplete dominance of B (B is dominant for $u = 1$, B is recessive for $u = 0$). As to males, we assume in model (4a) that B is recessive, and selection is sexually antagonistic, i.e.,

$$\mu_1 = \gamma < 1, \qquad \mu_2, \ldots, \mu_6 = 1,$$

while in model (5a) we assume overdominance for the allele B

$$\mu_1 = \gamma < 1, \qquad \mu_3 = \gamma' = 1.2, \qquad \mu_2 = \mu_4 = \mu_5 = \mu_6 = 1,$$

where the value of $\gamma' > 1$ is chosen sufficiently close to 1 since the heterozygote advantage, if present, is not expected to be large.

2.4 Two X-linked loci – model (4b)

Here N=10, M=4, n=4, m=1, since due to linkage we must distinguish between the genotypes AB/ab and Ab/aB. The female genotypes i,h=1,…,9, are: 1=$AABB$, 2=$AaBB$, 3=$aaBB$, 4=$AABb$, 5=$AaBb$, 6=$AabB$, 7=$aaBb$, 8=$AAbb$, 9=$Aabb$, 10=$aabb$, while the male genotypes labes are: h,k=1,…,4, with 1=A-B-, 2=a-B-, 3=A-b-, 4=a-b-. Also, $\alpha = 1,\ldots,4$ and the female gametes are 1=AB, 2=aB, 3=Ab, 4=ab; there is only one male gamete (- -) with β=1. As before, we view the first X-linked allele A as an activator of the GFMH associated to the allele B on the second X-linked locus. We assume incomplete dominance for the allele B in females, i.e.,

$$\psi_1 = \psi_2 = \alpha > 1, \qquad \psi_4 = \psi_5 = \psi_6 = 1 + u(\alpha - 1), \qquad \psi_3 = \psi_7 = \psi_8 = \psi_9 = \psi_{10} = 1,$$

and in males selection is antagonistic to females

$$\mu_1 = \gamma < 1, \qquad \mu_2 = \mu_3 = \mu_4 = 1.$$

2.5 Two autosomal loci – models (4c), (5b) and (7)

Here N=M=10 (since we must distinguish between the genotypes AB/ab and Ab/aB) and n=m=4, with i,j,h,k=1,…,10, for genotypes (1=$AABB$, 2=$AaBB$, 3=$aaBB$, 4=$AABb$, 5=$AaBb$, 6=$AabB$, 7=$aaBb$, 8=$AAbb$, 9=$Aabb$, 10=$aabb$) and $\alpha = 1,\ldots,4$, label gametes (1=AB, 2=aB, 3=Ab, 4=ab). As before, we view the first autosomal allele A as an activator of the GFMH associated to the second autosomal allele B.

– In model (4c) we assume antagonistic selection for males and females, and incomplete dominance of B in females:

$$\psi_1 = \psi_2 = \alpha > 1, \qquad \psi_4 = \psi_5 = \psi_6 = 1 + u(\alpha - 1), \qquad \psi_3 = \psi_7 = \psi_8 = \psi_9 = \psi_{10} = 1,$$

while in males we assume B to be recessive, with

$$\mu_1 = \mu_2 = \gamma < 1, \qquad \mu_3 = \ldots = \mu_{10} = 1.$$

– In model (5b) we still assume incomplete dominance of B in females, but overdominance for B in males,

$$\mu_1 = \mu_2 = \gamma < 1, \qquad \mu_4 = \mu_5 = \mu_6 = \gamma' = 1.2, \qquad \mu_3 = \mu_7 = \mu_8 = \mu_9 = \mu_{10} = 1.$$

– In model (7) for maternal effects, see formula (5), we again assume incomplete dominance for *B* in females, so that:

$$\rho_1 = \rho_2 = \gamma < 1, \qquad \rho_4 = \rho_5 = \rho_6 = 1 - u(1-\gamma), \qquad \rho_3 = \rho_7 = \rho_8 = \rho_9 = \rho_{10} = 1.$$

2.6 Two independent autosomal loci with genomic imprinting – model (6)

The natural choice for the allele subject to imprinting is *A*, which is assumed to be active only when inherited from the mother. In this case we have N=9 and M=10. In fact, by independence, we do not distinguish anymore between the genotypes *AB*/*ab* and *Ab*/*aB*, but split the male genotype *AB*/*aB* in two classes: *AB*(maternal)/*aB*(paternal) and *aB*(maternal)/*AB*(paternal). Notice that we do not distinguish, for instance, between *AB*(maternal)/*ab*(paternal) and *aB*(maternal)/*Ab*(paternal), since we assume that B is recessive in males, so that the latter two genotypes give the same phenotype. Also, $n=m=4$. In this case i,j=1,…,9, label female genotypes (1=*AABB*, 2=*AaBB*, 3=*aaBB*, 4=*AABb*, 5=*AaBb*, 6=*aaBb*, 7=*AAbb*, 8=*Aabb*, 9=*aabb)*, while h,k=1,…,10, for male genotypes (1=*AABB*, 2=*A(m)a(p)BB*, 3=*a(m)A(p)BB*, 4=*aaBB*, 5=*AABb*, 6=*AaBb*, 7=*aaBb*, 8=*AAbb*, 9=*Aabb*, 10=*aabb*). Finally, α=1,…,4, label female and male gametes (1=*AB*, 2=*aB*, 3=*Ab*, 4=*ab*). Assuming incomplete dominance for the allele *B* in females, we write

$$\psi_1 = \psi_2 = \alpha > 1, \qquad \psi_4 = \psi_5 = 1 + u(\alpha - 1), \qquad \psi_3 = \psi_6 = \psi_7 = \psi_8 = \psi_9 = 1.$$

As to males, we assume that GFMH-related allele *B* is recessive, and selection is sexually antagonistic:

$$\mu_1 = \mu_2 = \gamma < 1, \qquad \mu_3 = \ldots = \mu_{10} = 1.$$

3. Correlation matrices for the pedigree analysis

Bayes' theorem yields the conditional probabilities of parental genotypes given the offspring genotype, described by the matrices (m= mother, f = father, d = daughter, s= son)

$$M_{hi} = \Pr(m = h \mid d = i) = \sum_{k=1}^{M} \frac{\Pr(d = i \mid m = h, f = k)\Pr(m = h)P(f = k)}{\Pr(d = i)},$$

$$N_{ki} = \Pr(f = k \mid d = i) = \sum_{h=1}^{N} \frac{\Pr(d = i \mid m = h, f = k)\Pr(m = h)P(f = k)}{\Pr(d = i)},$$

$$P_{hj} = \Pr(m = h \mid s = j) = \sum_{k=1}^{M} \frac{\Pr(s = j \mid m = h, f = k)\Pr(m = h)P(f = k)}{\Pr(s = j)},$$

$$Q_{kj} = \Pr(f = k \mid s = j) = \sum_{h=1}^{N} \frac{\Pr(s = j \mid m = h, f = k)\Pr(m = h)P(f = k)}{\Pr(s = j)},$$

where Pr(d=i) (Pr(s=j)) is the probability that a daughter (son) has genotype i [j], and is just the frequency ξ_i [η_j] of genotype i before selection. At equilibrium $x_i = x'_i$ and $y_j = y'_j$, and we obtain

$$M_{hi} = \frac{\sum_{k=1}^{M} A_{ihk} x_h y_k}{\sum_{h=1}^{N}\sum_{k=1}^{M} A_{ihk} x_h y_k}, \qquad N_{ki} = \frac{\sum_{h=1}^{N} A_{ihk} x_h y_k}{\sum_{h=1}^{N}\sum_{k=1}^{M} A_{ihk} x_h y_k},$$

$$P_{hj} = \frac{\sum_{k=1}^{M} B_{jhk} x_h y_k}{\sum_{h=1}^{N}\sum_{k=1}^{M} B_{jhk} x_h y_k}, \qquad Q_{kj} = \frac{\sum_{h=1}^{N} B_{jhk} x_h y_k}{\sum_{h=1}^{N}\sum_{k=1}^{M} B_{jhk} x_h y_k}.$$

Analogously, we compute the conditional probabilities relating brothers' and sisters' genotypes (ss = sister, b = brother) at equilibrium:

$$R_{ij} = \Pr(ss = i \mid b = j) = \sum_{k=1}^{M}\sum_{h=1}^{N} \frac{\Pr(d = i \mid m = h, f = k)\Pr(s = j \mid m = h, f = k)\Pr(m = h)\Pr(f = k)}{\Pr(s = j)},$$

$$S_{ii'} = \Pr(ss = i \mid ss = i') = \sum_{k=1}^{M}\sum_{h=1}^{N} \frac{\Pr(d = i \mid m = h, f = k)\Pr(d = i' \mid m = h, f = k)\Pr(m = h)\Pr(f = k)}{\Pr(d = i')},$$

$$T_{ji} = \Pr(b = j \mid ss = i) = \sum_{k=1}^{M}\sum_{h=1}^{N} \frac{\Pr(d = i \mid m = h, f = k)\Pr(s = j \mid m = h, f = k)\Pr(m = h)\Pr(f = k)}{\Pr(d = i)},$$

$$W_{jj'} = \Pr(b = j \mid b = j') = \sum_{k=1}^{M}\sum_{h=1}^{N} \frac{\Pr(s = j \mid m = h, f = k)\Pr(s = j' \mid m = h, f = k)\Pr(m = h)\Pr(f = k)}{\Pr(s = j')},$$

obtaining

$$R_{ij} = \frac{\sum_{h=1}^{N}\sum_{k=1}^{M} A_{ihk} B_{jhk} x_h y_k}{\sum_{h=1}^{N}\sum_{k=1}^{M} B_{jhk} x_h y_k}, \qquad S_{ii'} = \frac{\sum_{h=1}^{N}\sum_{k=1}^{M} A_{ihk} A_{i'hk} x_h y_k}{\sum_{h=1}^{N}\sum_{k=1}^{M} A_{ihk} x_h y_k},$$

$$T_{ji} = \frac{\sum_{h=1}^{N}\sum_{k=1}^{M} A_{ihk} B_{jhk} x_h y_k}{\sum_{h=1}^{N}\sum_{k=1}^{M} A_{ihk} x_h y_k}, \qquad W_{jj'} = \frac{\sum_{h=1}^{N}\sum_{k=1}^{M} B_{jhk} B_{j'hk} x_h y_k}{\sum_{h=1}^{N}\sum_{k=1}^{M} B_{j'hk} x_h y_k},$$

which yield the conditional probabilities of maternal and paternal aunts' genotypes (ma = maternal aunts, pa = paternal aunts):

$$U_{ij} = \Pr(ma = i \mid s = j) = \sum_{i'=1}^{N} \Pr(ss = i \mid ss = i')\Pr(m = i' \mid s = j) = \sum_{i'=1}^{N} S_{ii'} P_{i'j},$$

$$V_{ij} = \Pr(pa = i \mid s = j) = \sum_{j'=1}^{M} \Pr(ss = i \mid b = j')\Pr(f = j' \mid s = j) = \sum_{i'=1}^{N} R_{ij'} Q_{j'j}.$$

A similar argument allows one to compute the correlation matrices between grandparents' and nephews' genotypes, or between maternal and paternal cousins and a given individual's genotype.

4. Outputs

In order to study the stability properties and pedigree asymmetries, we introduce the following four classes of indicators (outputs of each model), all viewed as functions of the normalized fecundities α and γ. Recall GFMH+ and GFMH– indicate GFMH-carriers and non-GFMH-carriers respectively, and denote by F and H the sets of genotypes for which $\psi_i = \alpha$ for $i \in F$, and $\mu_j = \gamma$ for $j \in H$ (these are the sets of GFMH-genotypes for females and for males, respectively).

(i) The equilibrium proportions η and ϕ respectively of GFMH+ males and GFMH+ females in the population, defined by

$$\eta = \eta(\alpha,\gamma) = \sum_{j\in H}\eta_{j,} \qquad \phi = \phi(\alpha,\gamma) = \sum_{i\in F}\xi_i \ , \tag{7}$$

where η_j and ξ_i are the equilibrium proportions of male and female genotypes j and i before selection (cf. (3)), for given α and γ. For the models based on maternal effects (see equation (5)), only the equilibrium proportion ϕ of GFMH+ females is relevant.

(ii) Proportion of GFMH+ males in the parental lines (the first two are not defined for $\eta = 0$, the second two when $1 - \eta = 0$):

– average proportion of GFMH+ maternal uncles and cousins of GFMH+ males

$$\sum_{j\in H}\sum_{p\in H}\left(\sum_{i=1}^{N}T_{ji}P_{ip} + \sum_{k=1}^{M}\sum_{h=1}^{N}\sum_{q=1}^{N}B_{jhk}S_{hq}P_{qp}y_k + \sum_{k=1}^{M}\sum_{h=1}^{N}\sum_{q=1}^{N}B_{jhk}T_{kq}P_{qp}x_h\right)\eta_p \Big/ \left(3\sum_{q\in H}\eta_q\right);$$

– average proportion of GFMH+ paternal uncles, paternal cousins, and fathers of GFMH+ males

$$\sum_{j\in H}\sum_{p\in H}\left(\sum_{i=1}^{M}W_{ji}Q_{ip} + Q_{jp} + \sum_{k=1}^{M}\sum_{h=1}^{N}\sum_{q=1}^{M}B_{jhk}R_{hq}Q_{qp}y_k + \sum_{k=1}^{M}\sum_{h=1}^{N}\sum_{q=1}^{M}B_{jhk}W_{kq}Q_{qp}x_h\right)\eta_p \Big/ \left(4\sum_{q\in H}\eta_q\right);$$

– average proportion of GFMH+ maternal uncles and cousins of GFMH– males

$$\sum_{j\in H}\sum_{p\notin H}\left(\sum_{i=1}^{N}T_{ji}P_{ip} + \sum_{k=1}^{M}\sum_{h=1}^{N}\sum_{q=1}^{N}B_{jhk}S_{hq}P_{qp}y_k + \sum_{k=1}^{M}\sum_{h=1}^{N}\sum_{q=1}^{N}B_{jhk}T_{kq}P_{qp}x_h\right)\eta_p \Big/ \left(3\sum_{q\notin H}\eta_q\right);$$

– average proportion of GFMH+ paternal uncles, paternal cousins, and fathers of GFMH– males

$$\sum_{j\in H}\sum_{p\notin H}\left(\sum_{i=1}^{M}W_{ji}Q_{ip} + Q_{jp} + \sum_{k=1}^{M}\sum_{h=1}^{N}\sum_{q=1}^{M}B_{jhk}R_{hq}Q_{qp}y_k + \sum_{k=1}^{M}\sum_{h=1}^{N}\sum_{q=1}^{M}B_{jhk}W_{kq}Q_{qp}x_h\right)\eta_p \Big/ \left(4\sum_{q\notin H}\eta_q\right).$$

(iii) Maternal fecundities: the normalized fecundity of the mother of a son with genotype j is

$$\boldsymbol{E}(\psi_m \mid s = j) = \sum_{k=1}^{N}\psi_k \Pr(m = k \mid s = j) = \sum_{k=1}^{N}\psi_k P_{kj},$$

so that the average fecundities of mothers of GFMH+ and GFMH– males are respectively:

$$\mathbf{E}(\psi_m \,|\, \text{son} \in H) = \sum_{j\in H}\sum_{k=1}^{N}\psi_k P_{kj}\eta_j \Big/ \sum_{q\in H}\eta_q, \qquad \mathbf{E}(\psi_m \,|\, \text{son} \notin H) = \sum_{j\notin H}\sum_{k=1}^{N}\psi_k P_{kj}\eta_j \Big/ \sum_{q\notin H}\eta_q \,.$$

(*iv*) Aunts' fecundities: since the expected fecundity of the aunt (maternal or paternal) of a nephew with genotype *j* is

$$\boldsymbol{E}(\psi_{ma} \,|\, s = j) = \sum_{k=1}^{N}\psi_k \Pr(ma = k \,|\, s = j) = \sum_{k=1}^{N}\psi_k U_{kj},$$
$$\boldsymbol{E}(\psi_{pa} \,|\, s = j) = \sum_{k=1}^{N}\psi_k \Pr(pa = k \,|\, s = j) = \sum_{k=1}^{N}\psi_k V_{kj},$$

we obtain:

– the fecundity of maternal aunts of GFMH+ males: $\mathbf{E}(\psi_{ma} \,|\, \text{s} \in H) = \sum_{j\in H}\sum_{k=1}^{N}\psi_k U_{kj}\eta_j \Big/ \sum_{q\in H}\eta_q$;

– the fecundity of paternal aunts of GFMH+ males: $\mathbf{E}(\psi_{pa} \,|\, \text{s} \in H) = \sum_{j\in H}\sum_{k=1}^{N}\psi_k V_{kj}\eta_j \Big/ \sum_{q\in H}\eta_q$;

– the fecundity of maternal aunts of GFMH– males: $\mathbf{E}(\psi_{ma} \,|\, \text{s} \notin H) = \sum_{j\notin H}\sum_{k=1}^{N}\psi_k U_{kj}\eta_j \Big/ \sum_{q\notin H}\eta_q$;

– the fecundity of paternal aunts of GFMH– males: $\mathbf{E}(\psi_{pa} \,|\, \text{s} \notin H) = \sum_{j\notin H}\sum_{k=1}^{N}\psi_k V_{kj}\eta_j \Big/ \sum_{q\notin H}\eta_q$.

In the Figures below, and in the main text, we also consider suitable ratios for the indicators (*ii*)-(*iv*), in order to better put in evidence the pedigree asymmetries in sexual orientation and fecundity.

A final important output of the models is the total fecundity increment of the population due to the GFMH, given by the difference between the fecundity of the actual population at equilibrium and the fecundity of a population with same baseline fecundity (which coincides with f_b defined earlier) but no GFMH+ genotypes:

$$\Delta f = \sum_{h=1}^{N}\sum_{k=1}^{M} f_h m_k \xi_h \eta_k - f_b \,.$$

This may also be written as

$$\Delta f = \frac{f_{GFMH}}{\alpha}\left\{\left[(\alpha-1)\phi+1\right]\left[(\gamma-1)\eta+1\right]-1\right\}.$$

If, for instance due to social or economic factors, the female fecundities f_b and f_{GFMH} decrease, there is a decrease of the total fecundity in the population. However, by (6), the normalized fecundity α of GFMH+ females is inversely proportional to the baseline fecundity f_b of GFMH– females, and if, as expected, f_{GFMH} decreases less than f_b, this produces an increase of α. As discussed in the main

text, Δf turns out to be an increasing function of α for the most relevant models, so that in conditions of falling female fecundities, the Δf due to the GFMH always opposes the fecundity reduction.

5. Results

Here we show the typical outputs of the models considered above, obtained by iterating numerically the dynamic equations in Section 1.

5.1 One autosomal locus with overdominance in males and direct selection in females – model (1b)

In this case overdominance guarantees the persistence of the GFMH for all values of the normalized fecundities in the range $1 < \alpha < 2$ and $0 < \gamma < 1$ (Figure 1A). However, the pedigree asymmetries are not sufficiently accounted for by this model. The graphs in Figure 1 show that, while there is a small increase of GFMH+ relatives in the parental lines of GFMH+ (red plots in Figures 1B-1D) with respect to parental lines of GFMH– (blue plots in Figures 1B-1D), there is virtually no difference in this respect between the maternal and paternal lines of both GFMH+ and GFMH– (dashed red and blue plots in Figures 1C-1E).

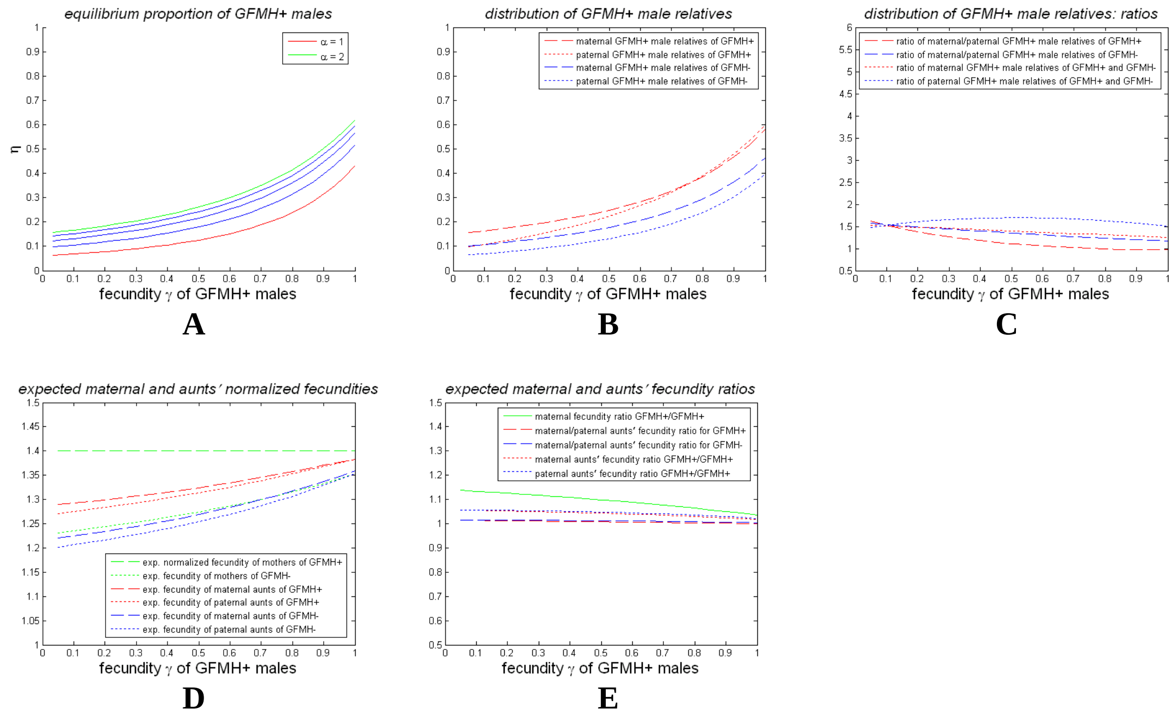

Figure 1. Plots for model (1b): one autosomal locus with overdominance in males. The blue plots in A correspond to values of α between 1 and 2, while $\alpha = 1.4$ in B, C, D, E.

5.2 One autosomal locus with antagonistic selection – model (1c)

Also in this case the GFMH remains stable for all values of the normalized fecundities in the range $1 < \alpha < 2$ and $0 < \gamma < 1$ (Figure 2A). Again, the pedigree asymmetries are not sufficiently accounted for by this model. However, the same conclusions of model (1b) hold here: there is a small increase of GFMH+ relatives in the parental lines of GFMH+ (Figures 2B-2D) but there is virtually no difference in this respect between the maternal and paternal lines of both GFMH+ and GFMH– (Figures 2C-2E).

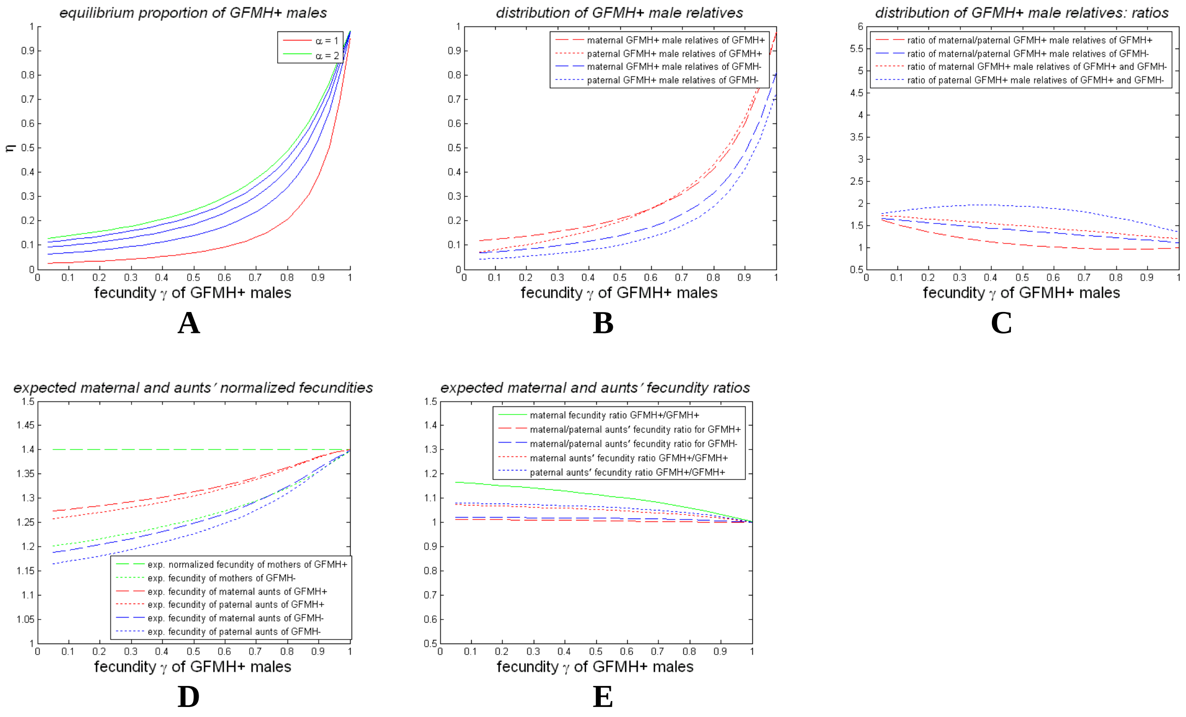

Figure 2. Plots for model (1c): one autosomal locus with sexually antagonistic selection. The blue plots in A correspond to values of α between 1 and 2, while $\alpha = 1.4$ in B, C, D, E.

5.3 One X-locus with antagonistic selection – model (2b)

This model is unstable in the range $1 < \alpha < 2$ and $0 < \gamma < 1$ (Figure 3A). The pedigree asymmetries, however, are well explained: the graphs in Figures 3B-3E show that there is a substantial increase of GFMH+ relatives in the maternal lines of GFMH+ (dashed red plots in Figures 3B-3D, and red plots in Figures 3C-3E), and there is virtually no difference between all other groups.

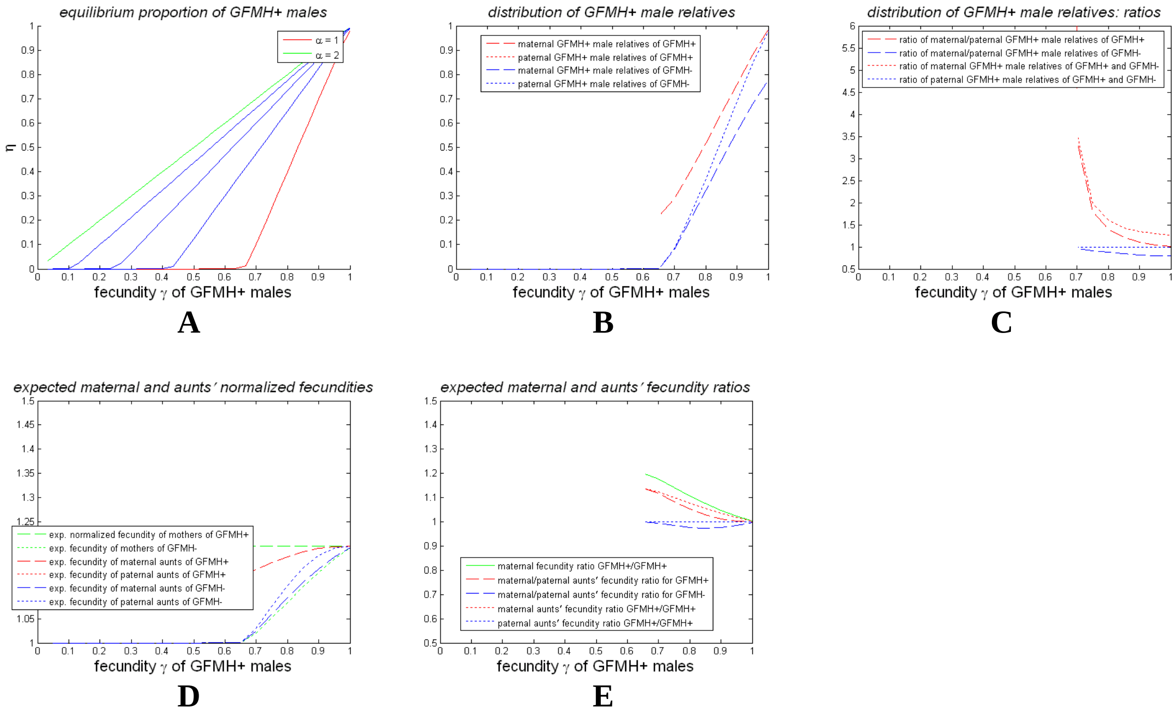

Figure 3. Plots for model (2b): one X-linked locus with sexually antagonistic selection. The blue plots in A correspond to values of α between 1 and 2, while $\alpha = 1.2$ in B, C, D, E.

5.4 One autosomal or X-linked locus with maternal selection – models (3a,b)

Both models are highly unstable in the range $1 < \alpha < 2$ and $0 < \gamma < 1$ (Figures 4A-4B).

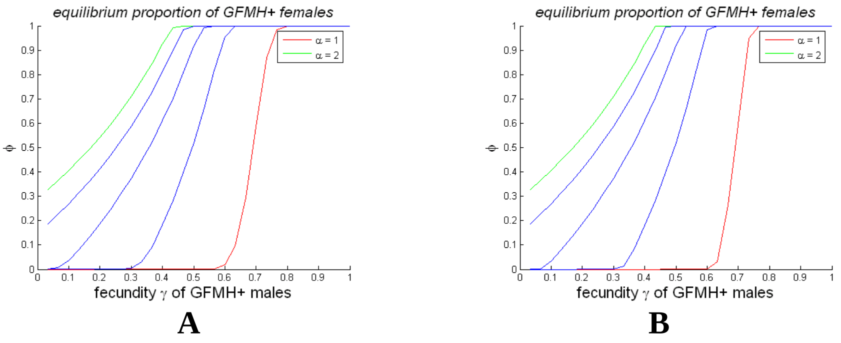

Figure 4. Plots for model (3a,b): an autosomal (A) and an X-linked locus (B) with maternal selection. The blue plots in A correspond to values of α between 1 and 2.

5.5 An X-linked locus and an autosomal locus with antagonistic selection – model (4a)

This model is stable in the range $1 < \alpha < 2$ and $0 < \gamma < 1$ (Figure 5A). The pedigree asymmetries are well explained: the graphs in Figure 5B-5E) show that there is a substantial increase of GFMH+ relatives in the maternal lines of GFMH+ (dashed red plots in Figures 5B-5D, and red plots in Figures 5C-5E), and there is virtually no difference between all other groups.

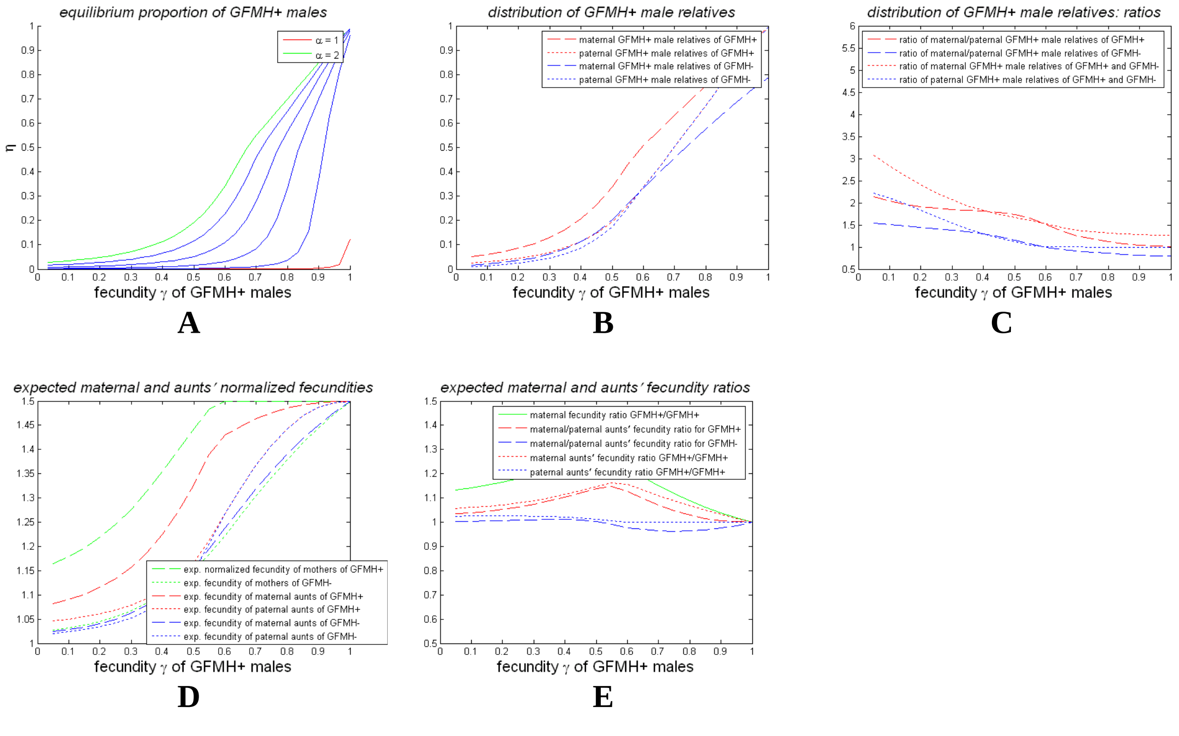

Figure 5. Plots for model (4a): one autosomal and one X-linked locus with sexually antagonistic selection. The blue plots in A correspond to values of α between 1 and 2, while $\alpha = 1.5$ and $u = 0.1$ in B, C, D, E.

5.6 Two X-linked loci with antagonistic selection – model (4b)

Again, this model is stable in the range $1 < \alpha < 2$ and $0 < \gamma < 1$ (Figure 6A), and the pedigree asymmetries are well explained: the graphs in Figures 6B-6E show that there is a large increase of GFMH+ relatives in the maternal lines of GFMH+ (dashed red plots in Figures 6B-6D, and red plots in Figure 6C-6E), and there is no difference between all other groups.

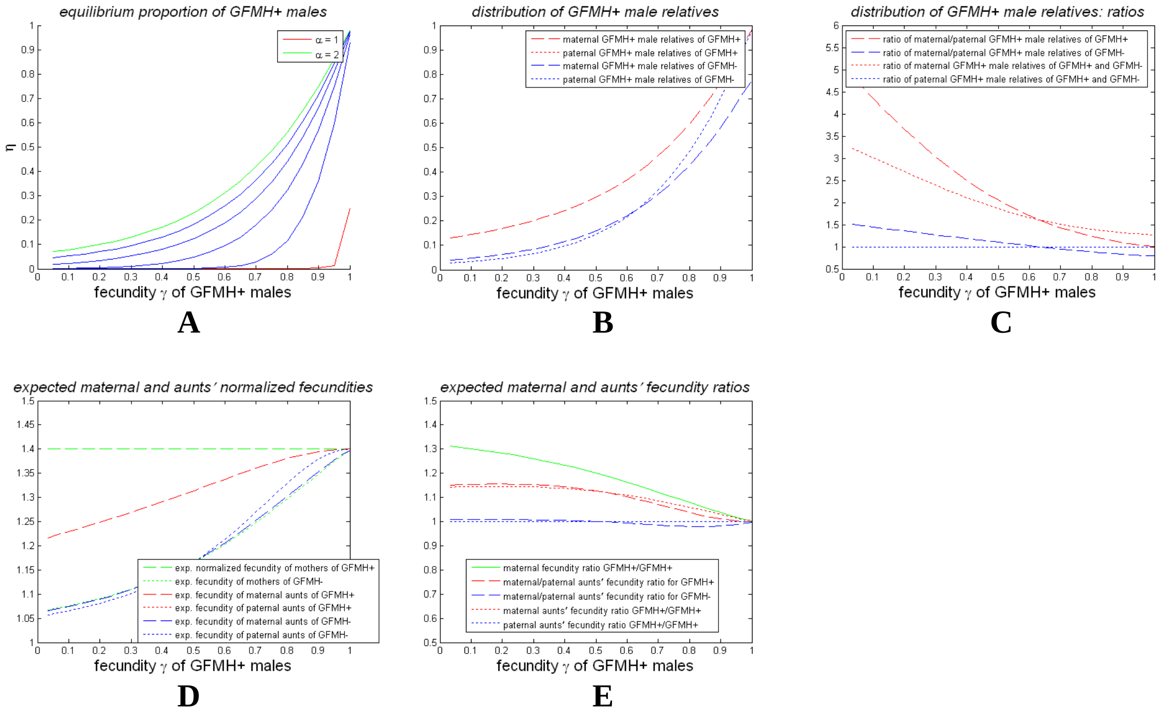

Figure 6. Plots for model (4b): two X-linked loci with sexually antagonistic selection. The blue plots in A correspond to values of α between 1 and 2, while $\alpha = 1.4$, $r = 0.25$, and $u = 1$ in B, C, D, E.

5.7 Two autosomal loci with antagonistic selection – model (4c)

There is no significant difference between this 2-locus model and its one-locus counterpart model (2c): the GFMH remains stable for all values of the normalized fecundities but pedigree asymmetries are not explained.

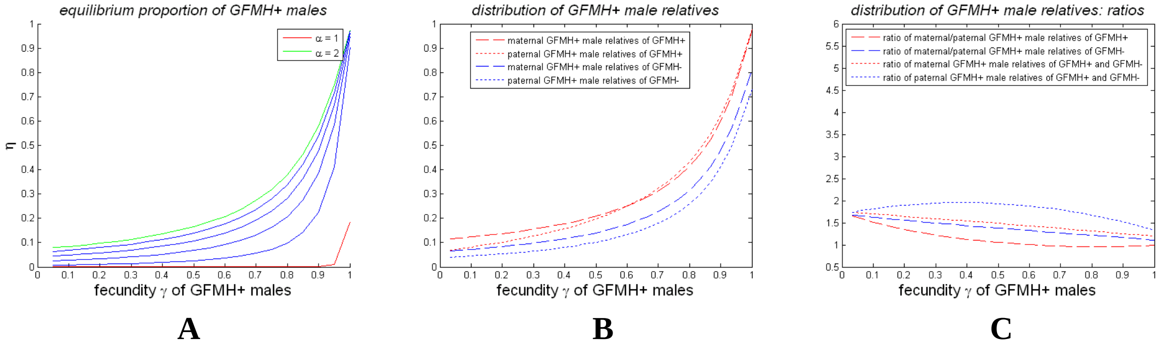

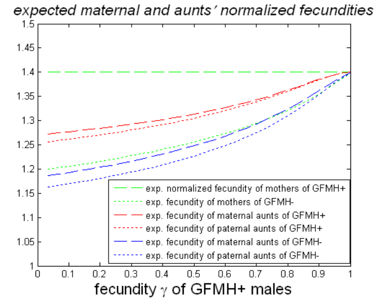

D

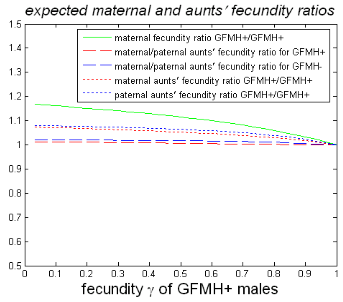

E

Figure 7. Plots for model (4c): two autosomal loci with sexually antagonistic selection. The blue plots in A correspond to values of α between 1 and 2, while α = 1.4, r = 0.25, and u = 1, in B, C, D, E.

5.8 An X-linked locus and an autosomal locus, with overdominance in males – model (5a)

A typical overdominance scenario as in model (1b). The GFMH is persistent for all values of the normalized fecundities in the range 1 < α < 2 and 0 < γ < 1. Again, the pedigree asymmetries are not sufficiently accounted for by this model: there is a small increase of GFMH+ relatives in the parental lines of GFMH+ with respect to parental lines of GFMH- but there is virtually no difference between the maternal and paternal lines of both GFMH+ and GFMH–.

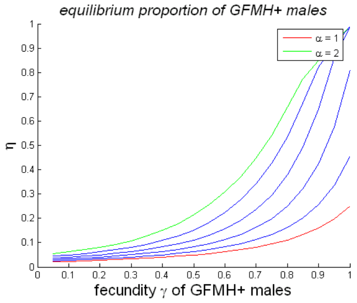

A

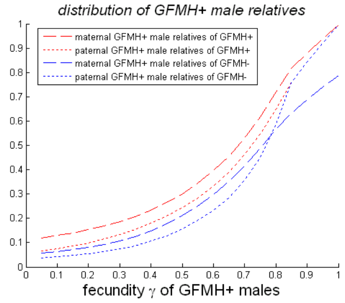

B

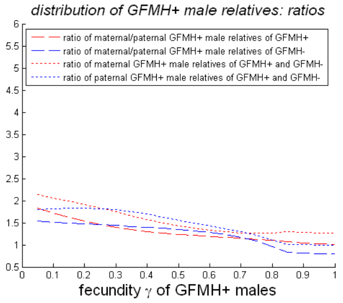

C

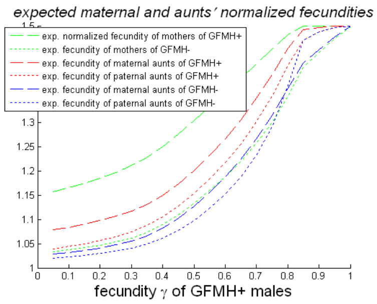

D

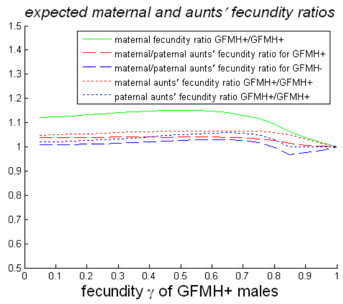

E

Figure 8. Plots for model (5a): one autosomal and one X-linked locus with overdominance in males. The blue plots in A correspond to values of α between 1 and 2, while α = 1.5 and u = 0 in B, C, D, E.

5.9 Two autosomal loci with overdominance in males – model (5b)

Again a typical overdominance scenario as in models (1b) and (5a).

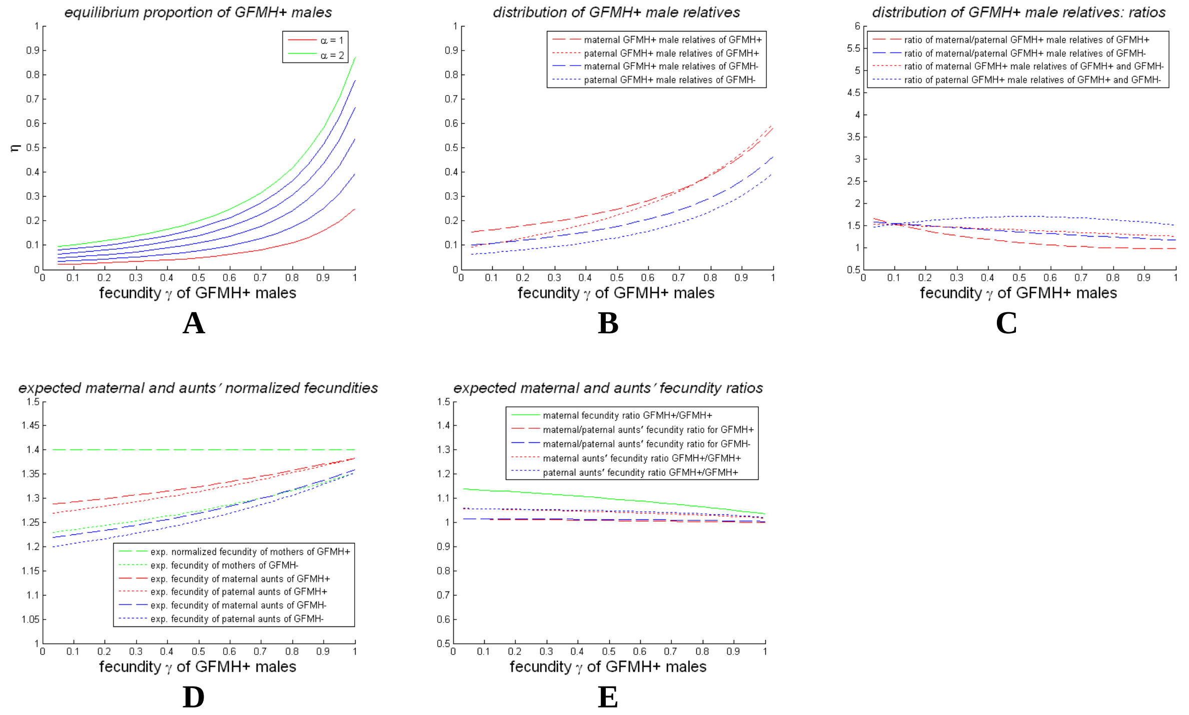

Figure 9. Plots for model (5b): two autosomal loci with overdominance in males. The blue plots in A correspond to values of α between 1 and 2, while $\alpha = 1.4$, $r = 0.25$, and $u = 1$ in B, C, D, E.

5.10 Two independent autosomal loci with genomic imprinting – model (6)

While this model is rather stable, it does not account well for pedigree asymmetries. Namely, while there is a small increase of GFMH+ relatives in the parental lines of GFMH+ (red plots in Figures 1B-1D) with respect to parental lines of GFMH- (blue plots in Figures 1B-1D), there is virtually no difference in this respect between the maternal and paternal lines of both GFMH+ and GFMH– (dashed red and blue plots in Figures 1C-1E).

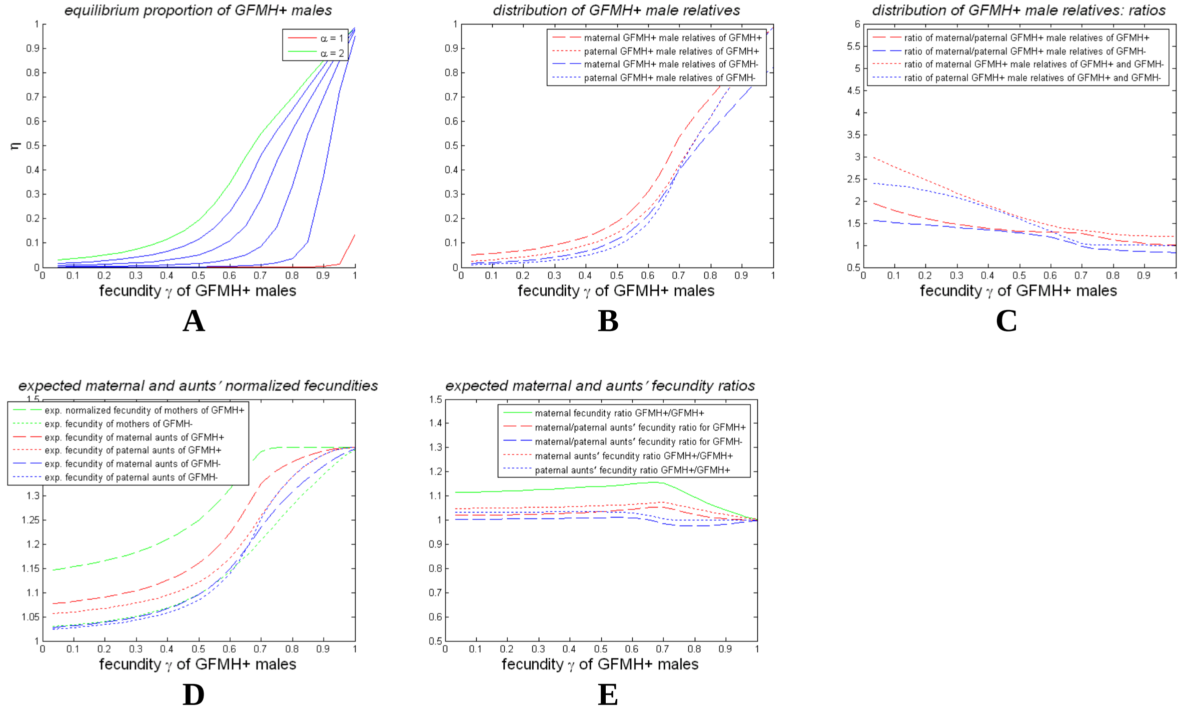

Figure 10. Plots for model (6): two autosomal loci with genomic imprinting. The blue plots in A correspond to values of α between 1 and 2, while $\alpha = 1.4$, $r = 0.25$, and $u = 0.2$ in B, C, D, E.

5.11 Two autosomal loci with maternal selection – model (7)

As for models (3a) and (3b), this model is highly unstable.

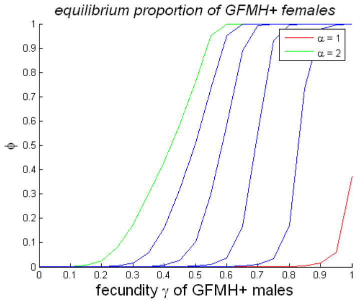

Figure 11. Plot for model (7): two autosomal loci with maternal selection. The blue plots in A correspond to values of α between 1 and 2, and $\alpha = 1.4$, $r = 0.25$, $u = 0.5$.

6. *Remarks on phenotypic expression*

As mentioned in the main text, the one- and two-locus models considered above for GFMH propagation fall into three groups, based respectively on the three different mechanisms of overdominance in males, maternal effects, and sexual antagonism. It is possible to relate each hypothesis to an interpretation in terms of the phenotypic expression of the GFMH.

(*i*) The hypothesis of expression of ‘maternal GFMH’, leads, on the one hand, to model (6) based on maternal genomic imprinting, and on the other hand, following the approach of Ref. [28], to models (3a), (3b), (7) based on genetic maternal effects on males.

(*ii*) The models based on overdominance in males (see (1b), (5a), (5b)) may be related to the hypothesis of expression of ‘feminizing GFMH’, as discussed for instance in [28]. In this case the GFMH induce feminization, increasing the probability of homosexuality in males, by directing the development towards the female sex determination. As indicated in [28], a plausible scenario for such feminizing GFMH would be overdominance in males, because such GFMH may give higher fitness to heterozygous males (who may for instance have higher success in attracting females). Feminizing GFMH, however, are always favorable to females, and would induce positive directional selection in females.

We notice the overdominance mechanism considered in (1b), (5a), (5b), does not coincide with the classical hypothesis in which overdominance is manifested in all carriers regardless of sex. Indeed, the feminizing GFMH, being always favorable to females, always introduces in the overdominance models considered here a sexually antagonistic component.

(*iii*) The models based on sexually antagonist selection (see (1c), (2b), (4a), (4b), (4c)), are naturally related to the hypothesis of expression of ‘androphilic GFMH’. By this we mean that the GFMH induce, rather than an overall feminization, a more focused attraction to males as a target of the mating behaviour. In this case the plausible scenario is negative directional selection in males with no heterozygote advantage. Androphilic GFMH however, are always favorable to females, and induce positive directional selection in females.

As a consequence of the above discussion, the conclusion (see the main text) that the GFMH have sexually antagonistic character, suggests an androphilic expression of the GFMH. We notice that this allows one, in principle, to make testable predictions regarding the behavior of both male and female GFMH carriers.

7. *List of the gamete-genotype correlation matrices*

7.1 A single autosomal locus

$$(C_{\alpha h}) = (D_{\alpha k}) = (E_{\beta k}) = \begin{pmatrix} 1 & 1/2 & 0 \\ 0 & 1/2 & 1 \end{pmatrix}, \quad (F_{1\alpha\alpha'}) = (G_{1\alpha\beta}) = \begin{pmatrix} 1 & 0 \\ 0 & 0 \end{pmatrix},$$
$$(F_{2\alpha\alpha'}) = (G_{2\alpha\beta}) = \begin{pmatrix} 0 & 1 \\ 1 & 0 \end{pmatrix}, \qquad (F_{3\alpha\alpha'}) = (G_{3\alpha\beta}) = \begin{pmatrix} 0 & 0 \\ 0 & 1 \end{pmatrix}.$$

7.2 A single X-linked locus

$$(C_{\alpha h}) = \begin{pmatrix} 1 & 1/2 & 0 \\ 0 & 1/2 & 1 \end{pmatrix} \quad (D_{\alpha k}) = \begin{pmatrix} 1 & 0 \\ 0 & 1 \end{pmatrix} \quad (E_{\beta k}) = \begin{pmatrix} 1 & 1 \end{pmatrix} \quad (G_{1\alpha\beta}) = \begin{pmatrix} 1 \\ 0 \end{pmatrix} \quad (G_{2\alpha\beta}) = \begin{pmatrix} 0 \\ 1 \end{pmatrix}$$
$$(F_{1\alpha\alpha'}) = \begin{pmatrix} 1 & 0 \\ 0 & 0 \end{pmatrix} \quad (F_{2\alpha\alpha'}) = \begin{pmatrix} 0 & 1 \\ 1 & 0 \end{pmatrix} \quad (F_{3\alpha\alpha'}) = \begin{pmatrix} 0 & 0 \\ 0 & 1 \end{pmatrix}.$$

7.3 Two loci: autosomal and X-linked

$$(C_{\alpha h}) = \begin{pmatrix} 1 & 1/2 & 0 & 1/2 & 1/4 & 0 & 0 & 0 & 0 \\ 0 & 1/2 & 1 & 0 & 1/4 & 1/2 & 0 & 0 & 0 \\ 0 & 0 & 0 & 1/2 & 1/4 & 0 & 1 & 1/2 & 0 \\ 0 & 0 & 0 & 0 & 1/4 & 1/2 & 0 & 1/2 & 1 \end{pmatrix} \quad (E_{\beta k}) = \begin{pmatrix} 1 & 1 & 1/2 & 1/2 & 0 & 0 \\ 0 & 0 & 1/2 & 1/2 & 1 & 1 \end{pmatrix} \quad (D_{\alpha k}) = \begin{pmatrix} 1 & 0 & 1/2 & 0 & 0 & 0 \\ 0 & 1 & 0 & 1/2 & 0 & 0 \\ 0 & 0 & 1/2 & 0 & 1 & 0 \\ 0 & 0 & 0 & 1/2 & 0 & 1 \end{pmatrix},$$

$$(F_{1\alpha\alpha'}) = \begin{pmatrix} 1&0&0&0 \\ 0&0&0&0 \\ 0&0&0&0 \\ 0&0&0&0 \end{pmatrix} \quad (F_{2\alpha\alpha'}) = \begin{pmatrix} 0&1&0&0 \\ 1&0&0&0 \\ 0&0&0&0 \\ 0&0&0&0 \end{pmatrix} \quad (F_{3\alpha\alpha'}) = \begin{pmatrix} 0&0&0&0 \\ 0&1&0&0 \\ 0&0&0&0 \\ 0&0&0&0 \end{pmatrix} \quad (F_{4\alpha\alpha'}) = \begin{pmatrix} 0&0&1&0 \\ 0&0&0&0 \\ 1&0&0&0 \\ 0&0&0&0 \end{pmatrix} \quad (F_{5\alpha\alpha'}) = \begin{pmatrix} 0&0&0&1 \\ 0&0&1&0 \\ 0&1&0&0 \\ 1&0&0&0 \end{pmatrix}$$
$$(F_{6\alpha\alpha'}) = \begin{pmatrix} 0&0&0&0 \\ 0&0&0&1 \\ 0&0&0&0 \\ 0&1&0&0 \end{pmatrix} \quad (F_{7\alpha\alpha'}) = \begin{pmatrix} 0&0&0&0 \\ 0&0&0&0 \\ 0&0&1&0 \\ 0&0&0&0 \end{pmatrix} \quad (F_{8\alpha\alpha'}) = \begin{pmatrix} 0&0&0&0 \\ 0&0&0&0 \\ 0&0&0&1 \\ 0&0&1&0 \end{pmatrix} \quad (F_{9\alpha\alpha'}) = \begin{pmatrix} 0&0&0&0 \\ 0&0&0&0 \\ 0&0&0&0 \\ 0&0&0&1 \end{pmatrix}.$$

and finally

$$(G_{1\alpha\beta}) = \begin{pmatrix} 1&0 \\ 0&0 \\ 0&0 \\ 0&0 \end{pmatrix} \quad (G_{2\alpha\beta}) = \begin{pmatrix} 0&0 \\ 1&0 \\ 0&0 \\ 0&0 \end{pmatrix} \quad (G_{3\alpha\beta}) = \begin{pmatrix} 0&1 \\ 0&0 \\ 1&0 \\ 0&0 \end{pmatrix} \quad (G_{4\alpha\beta}) = \begin{pmatrix} 0&0 \\ 0&1 \\ 0&0 \\ 1&0 \end{pmatrix} \quad (G_{5\alpha\beta}) = \begin{pmatrix} 0&0 \\ 0&0 \\ 0&1 \\ 0&0 \end{pmatrix} \quad (G_{6\alpha\beta}) = \begin{pmatrix} 0&0 \\ 0&0 \\ 0&0 \\ 0&1 \end{pmatrix}.$$

7.4 Two X-linked loci

$C_{\alpha h}$ and $F_{i\alpha\alpha'}$ are as in 7.5, and

$$(D_{\alpha k}) = \begin{pmatrix} 1&0&0&0 \\ 0&1&0&0 \\ 0&0&1&0 \\ 0&0&0&1 \end{pmatrix} \quad (E_{\beta k}) = \begin{pmatrix} 1 & 1 & 1 & 1 \end{pmatrix} \quad (G_{1\alpha\beta}) = \begin{pmatrix} 1 \\ 0 \\ 0 \\ 0 \end{pmatrix} \quad (G_{2\alpha\beta}) = \begin{pmatrix} 0 \\ 1 \\ 0 \\ 0 \end{pmatrix} \quad (G_{3\alpha\beta}) = \begin{pmatrix} 0 \\ 0 \\ 1 \\ 0 \end{pmatrix} \quad (G_{4\alpha\beta}) = \begin{pmatrix} 0 \\ 0 \\ 0 \\ 1 \end{pmatrix}.$$

7.5 Two autosomal loci

By setting r the recombination fraction, $\lambda = (1–2r)$ is the linkage coefficient.

$$\left(C_{\alpha h}\right)=\left(E_{\beta k}\right)=\left(D_{\alpha k}\right)=\begin{pmatrix} 1 & 1/2 & 0 & 1/2 & (1+\lambda)/4 & (1-\lambda)/4 & 0 & 0 & 0 & 0 \\ 0 & 1/2 & 1 & 0 & (1-\lambda)/4 & (1+\lambda)/4 & 1/2 & 0 & 0 & 0 \\ 0 & 0 & 0 & 1/2 & (1-\lambda)/4 & (1+\lambda)/4 & 0 & 1 & 1/2 & 0 \\ 0 & 0 & 0 & 0 & (1+\lambda)/4 & (1-\lambda)/4 & 1/2 & 0 & 1/2 & 1 \end{pmatrix},$$

$$\left(F_{1\alpha\alpha'}\right)=\left(G_{1\alpha\beta}\right)=\begin{pmatrix} 1 & 0 & 0 & 0 \\ 0 & 0 & 0 & 0 \\ 0 & 0 & 0 & 0 \\ 0 & 0 & 0 & 0 \end{pmatrix} \quad \left(F_{2\alpha\alpha'}\right)=\left(G_{2\alpha\beta}\right)=\begin{pmatrix} 0 & 1 & 0 & 0 \\ 1 & 0 & 0 & 0 \\ 0 & 0 & 0 & 0 \\ 0 & 0 & 0 & 0 \end{pmatrix} \quad \left(F_{3\alpha\alpha'}\right)=\left(G_{3\alpha\beta}\right)=\begin{pmatrix} 0 & 0 & 0 & 0 \\ 0 & 1 & 0 & 0 \\ 0 & 0 & 0 & 0 \\ 0 & 0 & 0 & 0 \end{pmatrix}$$

$$\left(F_{4\alpha\alpha'}\right)=\left(G_{4\alpha\beta}\right)=\begin{pmatrix} 0 & 0 & 1 & 0 \\ 0 & 0 & 0 & 0 \\ 1 & 0 & 0 & 0 \\ 0 & 0 & 0 & 0 \end{pmatrix} \quad \left(F_{5\alpha\alpha'}\right)=\left(G_{5\alpha\beta}\right)=\begin{pmatrix} 0 & 0 & 0 & 1 \\ 0 & 0 & 0 & 0 \\ 0 & 0 & 0 & 0 \\ 1 & 0 & 0 & 0 \end{pmatrix} \quad \left(F_{6\alpha\alpha'}\right)=\left(G_{6\alpha\beta}\right)=\begin{pmatrix} 0 & 0 & 0 & 0 \\ 0 & 0 & 1 & 0 \\ 0 & 1 & 0 & 0 \\ 0 & 0 & 0 & 0 \end{pmatrix}.$$

$$\left(F_{7\alpha\alpha'}\right)=\left(G_{7\alpha\beta}\right)=\begin{pmatrix} 0 & 0 & 0 & 0 \\ 0 & 0 & 0 & 1 \\ 0 & 0 & 0 & 0 \\ 0 & 1 & 0 & 0 \end{pmatrix} \quad \left(F_{8\alpha\alpha'}\right)=\left(G_{8\alpha\beta}\right)=\begin{pmatrix} 0 & 0 & 0 & 0 \\ 0 & 0 & 0 & 0 \\ 0 & 0 & 1 & 0 \\ 0 & 0 & 0 & 0 \end{pmatrix} \quad \left(F_{9\alpha\alpha'}\right)=\left(G_{9\alpha\beta}\right)=\begin{pmatrix} 0 & 0 & 0 & 0 \\ 0 & 0 & 0 & 0 \\ 0 & 0 & 0 & 1 \\ 0 & 0 & 1 & 0 \end{pmatrix}$$

$$\left(F_{10\alpha\alpha'}\right)=\left(G_{10\alpha\beta}\right)=\begin{pmatrix} 0 & 0 & 0 & 0 \\ 0 & 0 & 0 & 0 \\ 0 & 0 & 0 & 0 \\ 0 & 0 & 0 & 1 \end{pmatrix}$$

7.6 Two independent autosomal loci with genomic imprinting

$C_{\alpha k}$ and $F_{i\alpha\alpha'}$ are as in 7.3, and

$$\left(E_{\beta k}\right)=\left(D_{\alpha k}\right)=\begin{pmatrix} 1 & 1/2 & 1/2 & 0 & 1/2 & 1/4 & 0 & 0 & 0 & 0 \\ 0 & 1/2 & 1/2 & 1 & 0 & 1/4 & 1/2 & 0 & 0 & 0 \\ 0 & 0 & 0 & 0 & 1/2 & 1/4 & 0 & 1 & 1/2 & 0 \\ 0 & 0 & 0 & 0 & 0 & 1/4 & 1/2 & 0 & 1/2 & 1 \end{pmatrix},$$

$$\left(G_{1\alpha\beta}\right)=\begin{pmatrix} 1 & 0 & 0 & 0 \\ 0 & 0 & 0 & 0 \\ 0 & 0 & 0 & 0 \\ 0 & 0 & 0 & 0 \end{pmatrix} \quad \left(G_{2\alpha\beta}\right)=\begin{pmatrix} 0 & 1 & 0 & 0 \\ 0 & 0 & 0 & 0 \\ 0 & 0 & 0 & 0 \\ 0 & 0 & 0 & 0 \end{pmatrix} \quad \left(G_{3\alpha\beta}\right)=\begin{pmatrix} 0 & 0 & 0 & 0 \\ 1 & 0 & 0 & 0 \\ 0 & 0 & 0 & 0 \\ 0 & 0 & 0 & 0 \end{pmatrix} \quad \left(G_{4\alpha\beta}\right)=\begin{pmatrix} 0 & 0 & 0 & 0 \\ 0 & 1 & 0 & 0 \\ 0 & 0 & 0 & 0 \\ 0 & 0 & 0 & 0 \end{pmatrix} \quad \left(G_{5\alpha\beta}\right)=\begin{pmatrix} 0 & 0 & 1 & 0 \\ 0 & 0 & 0 & 0 \\ 1 & 0 & 0 & 0 \\ 0 & 0 & 0 & 0 \end{pmatrix}$$

$$\left(G_{6\alpha\beta}\right)=\begin{pmatrix} 0 & 0 & 0 & 1 \\ 0 & 0 & 1 & 0 \\ 0 & 1 & 0 & 0 \\ 1 & 0 & 0 & 0 \end{pmatrix} \quad \left(G_{7\alpha\beta}\right)=\begin{pmatrix} 0 & 0 & 0 & 0 \\ 0 & 0 & 0 & 1 \\ 0 & 0 & 0 & 0 \\ 0 & 1 & 0 & 0 \end{pmatrix} \quad \left(G_{8\alpha\beta}\right)=\begin{pmatrix} 0 & 0 & 0 & 0 \\ 0 & 0 & 0 & 0 \\ 0 & 0 & 1 & 0 \\ 0 & 0 & 0 & 0 \end{pmatrix} \quad \left(G_{9\alpha\beta}\right)=\begin{pmatrix} 0 & 0 & 0 & 0 \\ 0 & 0 & 0 & 0 \\ 0 & 0 & 0 & 1 \\ 0 & 0 & 1 & 0 \end{pmatrix} \quad \left(G_{10\alpha\beta}\right)=\begin{pmatrix} 0 & 0 & 0 & 0 \\ 0 & 0 & 0 & 0 \\ 0 & 0 & 0 & 0 \\ 0 & 0 & 0 & 1 \end{pmatrix}.$$
